# Polyhalogenated Carbazole Impairs Dopaminergic Neurons through Dysregulation of Liquid–Liquid Phase Separation in *Caenorhabditis elegans*

**DOI:** 10.1002/advs.202502486

**Published:** 2025-10-09

**Authors:** Yuhang Luo, Hongbo Xu, Pei Peng, Xinhe Lu, Qian Zhou, Ziqing Gao, Cheng Tang, Hongyan Yin, Ying Cai, Gaga Mahai, Zhiqiang Zhu, Zuojun Liu, Hanzeng Li, Shunqing Xu

**Affiliations:** ^1^ School of Environmental Science and Engineering Hainan University Haikou China; ^2^ School of Tropical Agriculture and Forestry Hainan University Haikou China; ^3^ Department of Critical Care Medicine Huazhong University of Science and Technology Union Wuhan China

**Keywords:** *Caenorhabditis elegans*, liquid–liquid phase separation (LLPS), polyhalogenated carbazoles, toxicology, unfolded protein responses

## Abstract

Polyhalogenated carbazoles (PHCZ) are emerging organic pollutants derived from a variety of natural and synthetic sources. While PHCZ have raised environmental concerns due to its persistence, bioaccumulation, and widespread distribution, its neuronal toxicity remains largely understudied. Here, the general and neuronal toxicity of PHCZ using the model organism *Caenorhabditis elegans* (*C. elegans*) is evaluated. It is found that PHCZ can induce significant dopaminergic neurodegeneration, in addition to exhibiting general toxicity affecting development and male gamete differentiation. Mechanistically, PHCZ promote liquid–liquid phase separation (LLPS) of the Parkinson's disease (PD)‐associated protein α‐synuclein (α‐syn), and reduces the fluidity of the resultant condensate both *in vitro and in viv* *o*. PHCZ disrupts general protein homeostasis and specifically activates the unfolded protein response in the ER (UPR^ER^) through the IRE‐1 signaling axis. Moreover, PHCZ impairs mitochondrial functions, providing another mechanistic link to neuronal degeneration. Thus, this study uncovers a hitherto unrecognized neuronal toxicity of PHCZ, which is partly attributed to their capacity to dysregulate LLPS and UPR^ER^, offering new insights into their potential health risks.

## Graphical abstract

Polyhalogenated carbazoles (PHCZ), persistent environmental contaminants, preferentially accumulate in neuronal tissues. Studies using *Caenorhabditis elegans* and human neuronal cell lines reveal that PHCZ induce dopaminergic neurodegeneration by promoting liquid–liquid phase separation of α‐synuclein, reducing condensate fluidity, impairing mitochondrial integrity, and activating endoplasmic reticulum stress responses. This newly identified neurotoxicity highlights environmental contributions to neurodegenerative diseases.

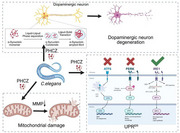

## 1 Introduction

Polyhalogenated carbazoles (PHCZs) are a group of persistent, bioaccumulative, and dioxin‐like compounds that have garnered increasing attention due to their environmental impact.^[^ ^1^, ^2^, ^3^, ^4^, ^5^ ^]^ PHCZs have been detected in water bodies worldwide, including lakes, rivers, and oceans.^[^ ^6^, ^7^, ^8^ ^]^ Notably, 11 types of PHCZs were identified in drinking water from Wuhan, China, with total concentrations reaching up to 53.48 ng L^−1^, highlighting the need for careful evaluation of the risks associated with PHCZs in water.^[^ ^9^ ^]^ The toxicological effects of PHCZs have been explored in several organisms, primarily utilizing aquatic animal models.^[^ ^10^ ^]^ These studies indicate that exposure to PHCZs leads to developmental abnormalities, cardiovascular toxicity, hepatotoxicity, and endocrine disruption.^[^ ^11^, ^12^, ^13^ ^]^ The neurotoxicity of PHCZ remain elusive. Evidence in *Carassius auratus* indicates that the brain exhibits the highest PHCZ enrichment among tissues.^[^ ^12^ ^]^ Early‐life exposure to 3,6‐Dibromocarbazole (3,6‐BCZ) in zebrafish significantly reduces the length and number of motor neuron axons, leading to impaired swimming ability and a weakened response to light stimuli.^[^ ^14^ ^]^ Furthermore, PHCZ detection in human urine samples raises concerns about potential neurotoxicity in humans,^[^ ^15^ ^]^ driven by their hydrophobic nature and resultant high bioaccumulative potential. Considering the environmental prevalence of PHCZ^[^ ^5^ ^]^ and detection in drinking water,^[^ ^9^ ^]^ there is an urgent need to deepen the understanding of PHCZ neurotoxicity and its underlying mechanisms.

Liquid–liquid phase separation (LLPS) is a biophysical process where biomolecules, such as proteins and nucleic acids, separate from the cytosolic pool to form dense, droplet‐like compartments within the cell.^[^ ^16^, ^17^, ^18^ ^]^ The occurrence of various diseases has been implicated because of abnormal LLPS of proteins, for example, TDP‐43,^[^ ^19^, ^20^ ^]^ α‐synuclein,^[^ ^21^, ^22^ ^]^ and DDX3X.^[^ ^23^, ^24^ ^]^ α‐synuclein, a neuronal protein associated with Parkinson's disease (PD), has been proved to undergo a LLPS and liquid‐to‐solid transition, to form an amyloid hydrogel that contains oligomers and fibrillar species, further intensifying its aggregation.^[^ ^21^ ^]^ The aggregation of α‐synuclein leads to the formation of Lewy bodies, which in turn contributes to the progression of PD.^[^ ^25^ ^]^ Environmental pollutants have also been shown to exacerbate α‐synuclein aggregation.^[^ ^26^, ^27^, ^28^ ^]^ Understanding the relation between environmental factors and α‐synuclein's LLPS behavior will offer new insights into the environmental etiology of PD as well as the dual role of LLPS in physiological cellular organization and pathological aggregation.

The endoplasmic reticulum (ER) is a critical organelle responsible for protein folding, lipid synthesis, and calcium homeostasis.^[^ ^29^, ^30^ ^]^ Proper functioning of the ER is essential for maintaining cellular homeostasis. Malfunctional ER leads to the accumulation of misfolded or unfolded proteins, a condition known as ER stress.^[^ ^31^ ^]^ To mitigate this stress, cells activate a protective mechanism called the unfolded protein response (UPR), which restores ER homeostasis by enhancing protein folding via chaperon upregulation, degrading misfolded proteins, and attenuating protein synthesis.^[^ ^32^, ^33^ ^]^ The UPR^ER^ is regulated by three parallel pathways: IRE‐1, which processes XBP‐1 mRNA; PERK, which phosphorylates eIF2α to upregulate ATF‐4; and ATF‐6, which undergoes cleavage to form an active transcription factor. These pathways mitigate ER stress and maintain cellular function, but prolonged activation may trigger apoptosis.^[^ ^34^ ^]^ Recent studies have shown that environmental pollutants, including heavy metals,^[^ ^35^, ^36^ ^]^ persistent organic pollutants,^[^ ^37^, ^38^ ^]^ and nanoparticles,^[^ ^39^ ^]^ can induce ER stress and UPR^ER^. Chronic exposure to such pollutants has been linked to various pathophysiological conditions, highlighting the significance of understanding ER stress as a key mediator of pollutant‐induced cellular toxicity. Mitochondria are also essential organelles responsible for energy production, regulation of cellular metabolism, and control of versatile signaling pathways in organisms.^[^ ^40^ ^]^ It plays a critical role in maintaining cellular homeostasis by generating ATP through oxidative phosphorylation and is central to various metabolic processes.^[^ ^41^ ^]^ Exposure to environmental pollutants, especially those can penetrate the cell and accumulate in mitochondria, has been shown to disrupt mitochondrial function, leading to impaired energy production, increased oxidative stress, and damage to mitochondrial DNA.^[^ ^42^, ^43^ ^]^ Such mitochondrial dysregulation results in adverse effects on reproduction and development.^[^ ^44^ ^]^ PHCZ has been shown to induce mitochondrial damage in zebrafish. Emerging contaminant 1,3,6,8‐tetrabromocarbazole induces oxidative damage and apoptosis during the embryonic development of zebrafish,^[^ ^45^ ^]^ further emphasizing the potential reproductive and developmental risks posed by mitochondrial‐targeting contaminants. However, PHCZ's neuronal toxicity and the underlying mechanisms were understudied. Here, we exposed the genetic model organism, *C. elegans* to PHCZ and found a dopaminergic neurodegenerative phenotype even at environmentally relevant concentrations, which involves mechanisms of LLPS dysregulation. These findings exemplify how organic contaminants disrupt LLPS and recapitulate the environmental risk in PD pathogenesis.

## 2 Results

### 2.1 General Toxicity Induced by PHCZ in *C. elegans*

**Figure 1:**
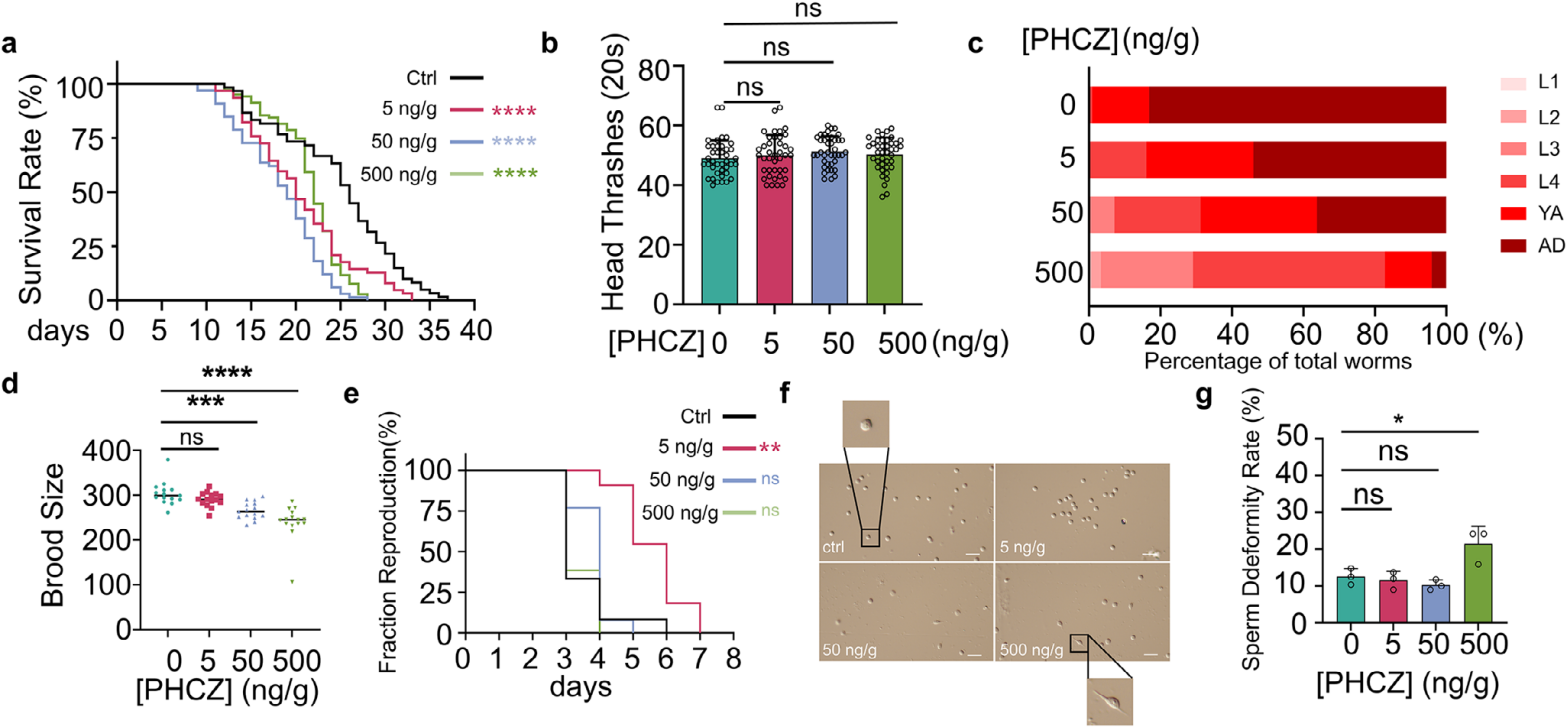
PHCZ causes dose‐dependent toxicity to both development and reproduction in *C. elegans*. a) Lifespan of wild‐type *C. elegans* that were exposed to PHCZ with indicated concentrations. (*n* = 60 (Ctrl), 62 (5 ng g^−1^), 66 (50 ng g^−1^), 103 (500 ng g^−1^). Pairwise log‐rank (Mantel–Cox) versus control: 5 ng g^−1^ *χ* ^2^ = 13.82, *p *= 2 × 10^−^⁴; 50 ng g^−1^ *χ* ^2^ = 47.63, *p *= 5.2 × 10^−12^; 500 ng g^−1^ *χ* ^2^ = 33.52, *p *= 7.1 × 10^−^⁹). b) Head thrash frequency of worms on day 1 of adulthood (*n *= 40 per group. Welch *t* (vs control, Holm‐adjusted): 5 ng g^−1^ *t* (77) = 0.71, *p *= 0.648, *g *= 0.16; 50 ng g^−1^ *t* (75.6) = 1.9, *p *= 0.183, *g *= 0.42; 500 ng g^−1^ *t* (77.6) = 0.99, *p *= 0.648, *g *= 0.22). c) Percentages of worms at each developmental stage after PHCZ exposure with indicated concentrations at 72 h post hatch (chi‐square (2 × k), Benjamini–Hochberg FDR correction; effect size is Cramér's V. Sample sizes (total worms): *n* = 119 (Ctrl), 146 (5 ng g^−1^), 141 (50 ng g^−1^), 244 (500 ng g^−1^). Results—Control versus 500 ng g^−1^: *χ* ^2^(5) = 263.18, *N* = 363, FDR *p* = 2.45 × 10^−^⁵⁴, *V* = 0.85; Control versus 50 ng g^−1^: *χ* ^2^(3) = 66.3, *N* = 260, FDR *p* = 3.96 × 10^−1^⁴, *V* = 0.50; Control versus 5 ng g^−1^: *χ* ^2^(2) = 29.89, *N* = 265, FDR *p* = 3.23 × 10^−^⁷, *V *= 0.34.). d) Brood size of worms with indicated treatments. (*n *= 15 per group). Welch *t* versus control (Holm‐adjusted): 5 ng g^−1^ *t* (23) = −1.64, *p* = 0.114, *g* = −0.58; 50 ng g^−1^ *t* (25.5) = −4.56, *p* = 2.22 × 10^−^⁴, *g* = −1.62; 500 ng g^−1^ *t* (23.9) = −5.13, *p* = 8.95 × 10^−^⁵, *g* = −1.82.) e) Reproductive spans of worms. (Pairwise vs Ctrl (Holm‐adjusted): 5 ng g^−1^ *χ* ^2^ = 16.79, *p *= 0.0064; 50 ng g^−1^ *χ* ^2^ = 7.24, *p *= 0.129; 500 ng g^−1^ *χ* ^2^ = 1.46, *p *= 0.481.) f) Representative images of sperms isolated from male worms with indicated treatments. g) Quantification of malformed sperm (Dunnett's (vs control): 5 ng g^−1^ Δmean = 0.987 [95% CI −6.026, 7.999], *padj* = 0.955; 50 ng g^−1^ Δmean = 2.297 [95% CI −4.715, 9.310], *padj* = 0.679; 500 ng g^−1^ Δmean = −8.873 [95% CI −15.89, −1.861], *padj* = 0.0165). Scale bars represent 100 µm in panel (f). Data were shown as mean ± SEM in (b,d,g). ns: not significantly different; *: *p *< 0.05; **: *p *< 0.01; ***: *p *< 0.001; ****: *p *< 0.0001.

The environmental concentration of PHCZ varies across regions and seasons, ranging from ≈10 to 41,362 ng g^−1^.^[^ ^46^, ^47^, ^48^, ^49^ ^]^ We set out to assess the general toxicities of PHCZ at environmentally relevant concentrations. Notably, exposure to 5–500 ng g^−1^ PHCZ resulted in a significant reduction in the lifespan of *C. elegans* (**Figure** **1**a), although overall muscle function remained largely unaffected (Figure 1b; Figure S1a, Supporting Information). Furthermore, PHCZ treatment caused significant developmental delays in *C. elegans* (Figure 1c). Besides the developmental toxicity, we also observed that exposure to PHCZ at concentrations as low as 50 ng g^−1^ led to a reduced brood size in *C. elegans* (Figure 1d). Consistent with this, RNA‐seq analysis revealed an abnormal upregulation of genes involved in spermiogenesis (Figure S1b,c, Supporting Information). Meanwhile, the male rates, often regarded as a marker for chromatin mis‐segregation, among the offspring of PHCZ‐exposed worms remain unaltered (Figure S1d, Supporting Information). Interestingly, the reproductive span of worms treated with 5 ng g^−1^ PHCZ was prolonged (Figure 1e), possibly indicating a disruption in the regulation of reproductive aging. Further examination of sperm function revealed a higher incidence of deformed sperm morphology in PHCZ‐treated males, characterized by spiked pseudopod structures, which resemble phenotypes of sperm overactivation (Figure 1f,g). When we tested sperm activation in vitro, sperm from PHCZ‐treated males exhibited higher activation potential compared to control males (Figure S1e, Supporting Information), implying a possible exhaustion of activation capacity due to PHCZ exposure. These results indicated that the reduced brood size was attributable to defects in sperm rather than oocytes.

### 2.2 PHCZ‐Mediated Developmental Toxicity is Independent of the Aryl Hydrocarbon Receptor (AHR)

**Figure 2:**
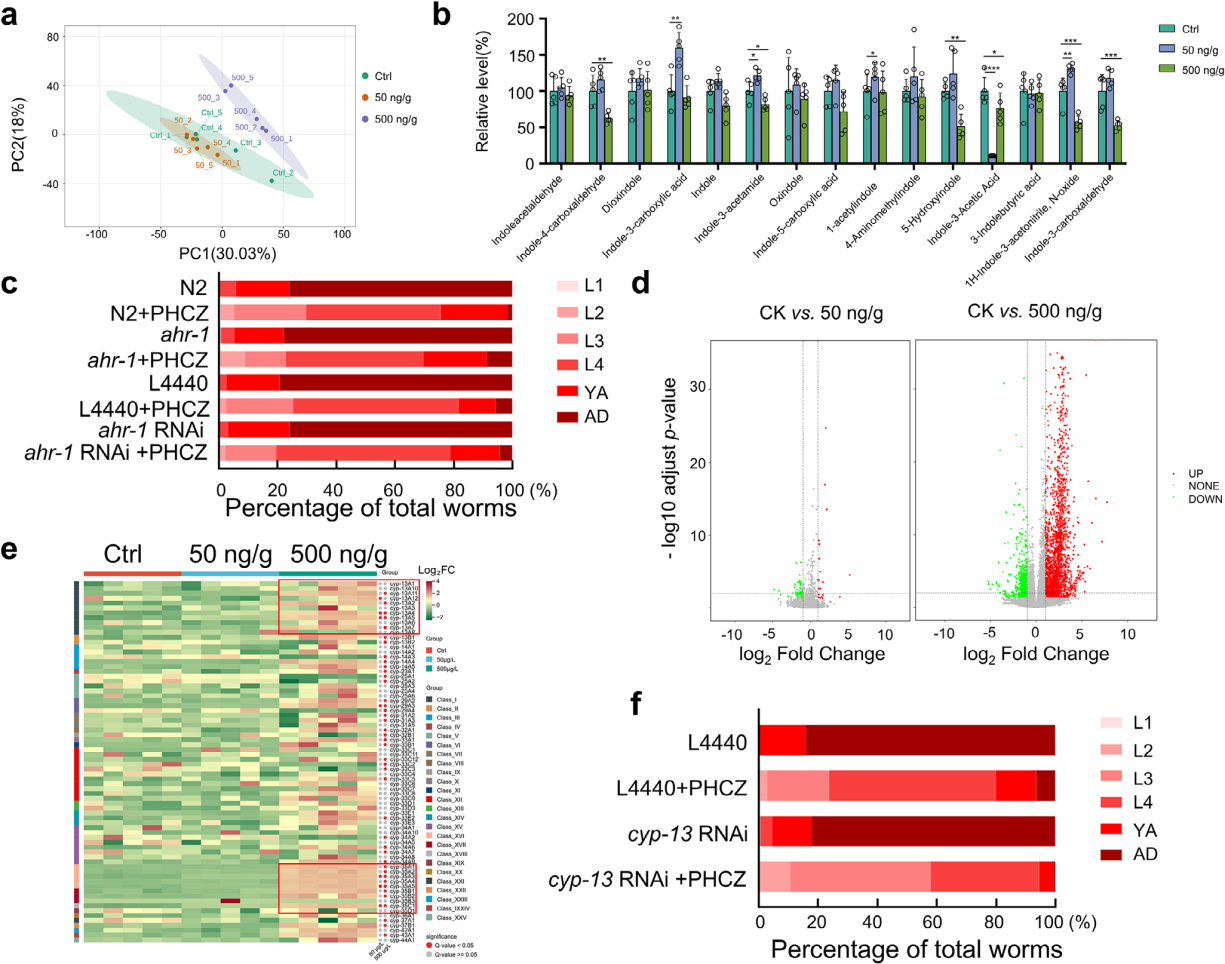
The developmental toxicity of PHCZ is not dependent on AHR‐1, but it does require the cytochrome c detoxifying enzyme family CYP‐13. a) Principal component analysis (PCA) of metabolic profiles in *C. elegans*. b) Relative metabolic levels of indole and its derivatives in *C. elegans* with 50 and 500 ng g^−1^ PHCZ treatments. The statistic values were shown in Table S5 (Supporting Information). c) Percentage of worms (with indicated treatment and genotypes) at each developmental stage at 72 h post hatch. Genotypes include *ahr‐1* loss‐of‐function mutant (see Experimental Section), *ahr‐1* RNAi, and control RNAi. PHCZ concentration in this experiment was 500 ng g^−1^. (Chi‐square tests of independence (2 × k). *p*‐values BH–FDR‐corrected across four comparisons; effect size = Cramér's V.Results— L4440 versus L4440+PHCZ: *χ* ^2^(4) = 441.44, *N* = 632, FDR *p *= 1.23 × 10^−^⁹^3^, *V* = 0.836; *ahr‐1* RNAi versus *ahr‐1* RNAi+PHCZ: *χ* ^2^(4) = 434.04, *N *= 645, FDR *p *= 2.45 × 10^−^⁹^2^, *V* = 0.820; N2 versus N2+PHCZ: *χ* ^2^(5) = 352.76, *N* = 545, FDR *p *= 4.45 × 10^−^⁷⁴, *V* = 0.805; *ahr‐1* versus *ahr‐1*+PHCZ: *χ* ^2^(5) = 419.84, *N* = 751, FDR *p *= 2.09 × 10^−^⁸⁸, *V* = 0.748. Totals (worms, control/PHCZ): L4440 334/298; *ahr‐1* RNAi 328/317; N2 312/233; *ahr‐1* 382/369) d) DEGs in 50 and 500 ng g^−1^ PHCZ treatments compared to the control visualized with a volcano plot. An adjusted *p‐*value < 0.05 and log_2_FoldChange > 2 was applied to define significantly up‐ or downregulated genes (red and green dots, respectively) or genes without change in expression (grey dots). e) Heatmap showing the relative abundance of cytochrome P450 family genes. The color scale indicates high (red) to low (green) abundance. f) Percentage of worms at each developmental stage at 72 h. Worms were fed with HT115 expressing *cyp‐13* or control RNAi, while PHCZ concentration was 500 ng g^−1^. (Chi‐square tests of independence (2 × k). *p*‐values BH–FDR–corrected across two comparisons; effect size = Cramér's V.Results— L4440 versus L4440+PHCZ: *χ* ^2^(4) = 276.62, *N* = 444, FDR *p* = 1.19 × 10^−^⁵⁸, *V* = 0.789; *cyp‐13* versus *cyp‐13*+PHCZ: *χ* ^2^(5) = 423.72, *N* = 508, FDR *p* = 4.57 × 10^−^⁸⁹, *V *= 0.913. Totals (worms, control/PHCZ): L4440 101/343; *cyp‐13* 178/330.) Data were shown as mean ± SEM in b). ns: not significantly different; *: *p *< 0.05; **: *p *< 0.01; ***: *p *< 0.001; ****: *p *< 0.0001.

The prevailing hypothesis regarding the mechanism of PHCZ action posits that it activates the AHR (with *ahr‐1* being the homolog in *C. elegans*),^[^ ^2^ ^]^ leading to adverse downstream effects. AHR signaling is tightly regulated and plays a key role in tuning metabolism. We thus performed metabolism analysis via mass spectrometry (Table S1, Supporting Information). The metabolic profiling revealed that PHCZ induces abnormal changes in the abundance of indole and its derivatives (**Figure** **2**a,b). For example, treatment with 50 ng g^−1^ PHCZ resulted in approximately a tenfold reduction in indole‐3‐acetic acid levels. However, *ahr‐1* RNAi and *ahr‐1* loss‐of‐function mutation did not alleviate the developmental retardation associated with PHCZ treatment (Figure 2c), suggesting that *ahr‐1* is not essential for PHCZ‐induced developmental toxicity. To comprehensively identify the alternative pathways involved in PHCZ toxicity, we delved into the PHCZ‐induced transcriptomic changes and identified 73 and 3217 differentially expressed genes (DEGs) in 50 and 500 ng g^−1^, respectively (Tables S2–S4, Supporting Information). As anticipated, treatment with 50 ng g^−1^ PHCZ resulted in only mild perturbations of bulk transcription in *C. elegans* (22 upregulated DEGs; 51 downregulated DEGs), whereas exposure to 500 ng g^−1^ PHCZ led to significantly more pronounced transcriptional dysregulation (2126 upregulated; 1091 downregulated, Figure 2d).

Analysis of the overlapping DEGs at both PHCZ concentrations revealed transcriptional activation of detoxification enzymes from the cytochrome P450 (CYP) family, specifically *cyp‐13* and *cyp‐35*, suggesting their role in the detoxification response to PHCZ toxicity (Figure 2e; Figure S2a–c, Tables S2–S4, Supporting Information). To confirm the role of *cyp‐13* in the detoxification process, we knocked down *cyp‐13* in *C. elegans* using the feeding RNAi method. The results showed that while *cyp‐13* knockdown did not affect the development of control (sham‐treated) worms, it significantly exacerbated the developmental defects induced by PHCZ exposure (Figure 2f). This indicates that worms deficient in *cyp‐13* are more susceptible to PHCZ. Overall, these findings imply that environmentally relevant concentrations of PHCZ may pose health risks and that the detoxification response does not involve the *ahr‐1* receptor‐mediated signaling pathway as previously expected.

### 2.3 PHCZ Leads to dopaminergic neuronal loss and Promotes Pathological Condensation of α‐Synuclein

**Figure 3:**
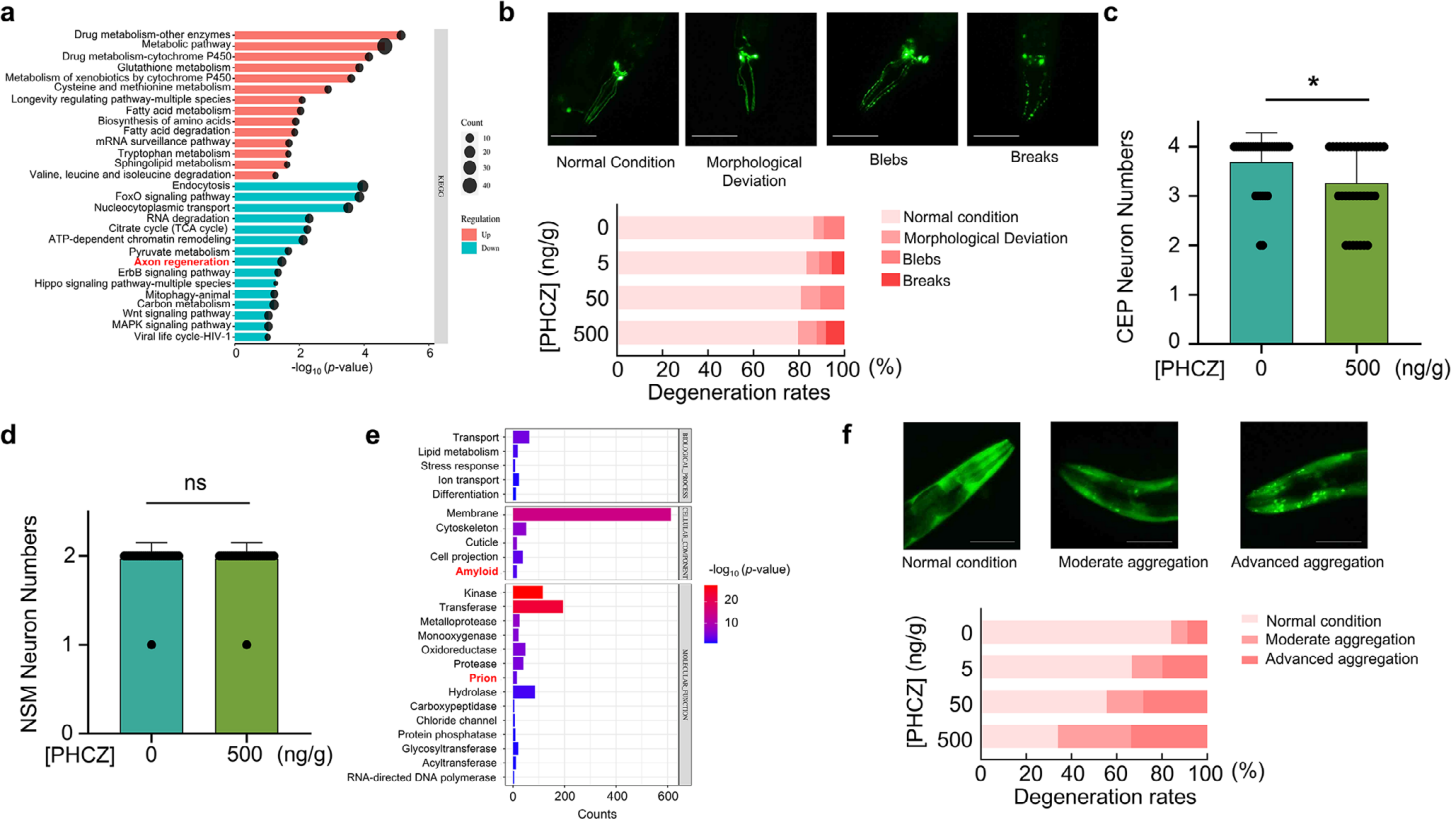
PHCZ specifically induces dopaminergic neuron degeneration and accelerates aggregation of α‐synuclein in *C. elegans*. a) KEGG pathway enrichment analysis in *C. elegans* exposed to 500 ng g^−1^ PHCZ. b) (Top) Representative fluorescence images of dopaminergic neurons in untreated controls and 500 ng g^−1^ PHCZ‐treated worms. Damage is indicated by morphological deviation, blebs, and breaks. (Bottom) Quantification of neuronal damage based on the percentage of worms with damaged neurons (Chi‐square tests of independence (2 × k). *p*‐values BH–FDR–corrected across three comparisons; effect size = Cramér's V.Results— 0 ng g^−1^ versus 5 ng g^−1^: *χ* ^2^(3) = 2.94, *N* = 98 (0 ng g^−1^ = 44, 5 ng g^−1^ = 54), FDR *p *= 0.601, *V* = 0.17; 0 ng g^−1^ versus 50 ng g^−1^: *χ* ^2^(2) = 0.68, *N* = 91 (0 ng g^−1^ = 44, 50 ng g^−1^ = 47), FDR *p *= 0.712, *V* = 0.09; 0 ng g^−1^ versus 500 ng g^−1^: *χ* ^2^(3) = 5.09, *N* = 93 (0 ng g^−1^ = 44, 500 ng g^−1^ = 49), FDR *p *= 0.496, *V* = 0.23.). c) Quantification of intact dopaminergic CEP neurons in day 7 of the adult UA44 strain, that ectopically express α‐synuclein under the control of the *dat‐1* promoter. (Unpaired *t*‐test (two‐sided). 0 ng g^−1^ (*n *= 99) versus 500 ng g^−1^ (*n *= 106): *t* (203) = 2.27, *p *= 0.024; Cohen's *d *= 0.32 (Hedges’ *g *= 0.32).) d) Quantification of intact serotonergic neurons in day 7 of adult GR1333 strain that express GFP under control of *tph‐1* promoter. e) GO enrichment analysis of upregulated genes in *C. elegans* exposed to 500 ng g^−1^ PHCZ. (Chi‐square test of independence (2 × 2) on two‐category scores; BH–FDR across one comparison (same as raw *p*). Effect size = Cramér's *V* (*φ*). Results— 0 ng g^−1^ versus 500 ng g^−1^: *χ* ^2^(1) = 0, *N* = 60 (0 ng g^−1^ = 30, 500 ng g^−1^ = 30), FDR *p* = 1, *V* = 0.) f) (Top) Representative image illustrating the three distinct states of α‐synuclein in *C. elegans*: normal condition, moderate aggregation, and advanced aggregation. (Bottom) Quantification of three kinds of worms (Chi‐square tests of independence (2 × 3) on aggregated category counts; *p*‐values BH–FDR–corrected across three comparisons; effect size = Cramér's V.Results— 0 ng g^−1^ versus 5 ng g^−1^: *χ* ^2^(2) = 5.86, *N* = 106 (CK = 57, 5 ng g^−1^ = 49), FDR *p *= 0.263, *V* = 0.235; 0 ng g^−1^ versus 50 ng g^−1^: *χ* ^2^(2) = 8.02, *N* = 102 (0 ng g^−1^ = 57, 50 ng g^−1^ = 45), FDR *p *= 0.132, *V* = 0.280; CK versus 500 ng g^−1^: *χ* ^2^(2) = 16.17, *N* = 104 (0 ng g^−1^ = 57, 500 ng g^−1^ = 47), FDR *p *= 0.0017, *V* = 0.395.). Scale bars represent 100 µm in (b,f). Data are shown as mean ± SEM in (c,d). ns: not significantly different; *: *p *< 0.05; **: *p *< 0.01; ***: *p *< 0.001; ****: *p *< 0.0001.

The RNA‐seq analysis identified enrichment in pathways related to axon regeneration (**Figure** **3**a), suggesting potential neurotoxicity of PHCZ, which has not been reported previously. Given that the neuronal lineages of *C. elegans* are invariant under normal conditions, we investigated the impact of PHCZ on different neuron types based on their neurotransmitters, that is, dopaminergic and serotonergic neurons. We began by quantifying the capacity of PHCZ in inducing neuronal cell death across these categories. Dopaminergic neurons (specifically CEP neurons in *C.elegans*, see Experimental Section) exhibited a mild loss of cell bodies and displayed morphological abnormalities in axons, such as blebbing and breaks, in worms treated with PHCZ at a concentration of 500 ng g^−1^ (Figure 3b,c). In contrast, serotonergic neurons (e.g., NSM neurons in worms, see Experimental Section) remained morphologically intact after PHCZ treatment (Figure 3d). Consistently, PHCZ exposure significantly increased calcein‐AM fluorescence while reducing Ki67‐positive cells in SH‐SY5Y cells, an immortalized human neuronal cell line, indicating suppressed proliferation and altered membrane permeability (Figure S3a–d, Supporting Information).

Although the exact etiology of PD is not fully delineated, abnormal aggregation of α‐synuclein, a pathological form of LLPS is believed to play a critical role in its pathogenesis. While the *C. elegans* homolog of α‐synuclein has not yet been identified, our transcriptional analysis revealed that PHCZ‐specific DEGs were notably enriched in amyloid and prion pathways (Figure 3e). To investigate whether PHCZ promotes α‐synuclein condensation, we treated a *C.elegans* transgenic strain expressing α‐synuclein::EGFP in muscle cells with PHCZ. The treatment resulted in a significant increase in the formation of α‐synuclein condensates during aging (Figure 3f). Similarly, a transgenic *C.elegans* strain ectopically expressing human‐derived α‐synuclein in dopaminergic neurons (see Experimental Section) exhibits accelerated aging‐dependent degeneration upon PHCZ treatment (Figure 3c), consistent with the results in Figure 3b. These findings suggest that PHCZ may contribute to neuronal degeneration by promoting the pathological condensation of both human‐derived α‐synuclein and the *C.elegans* counterparts, which are capable of undergoing LLPS and subsequent fibrotic solidification.

**Figure 4:**
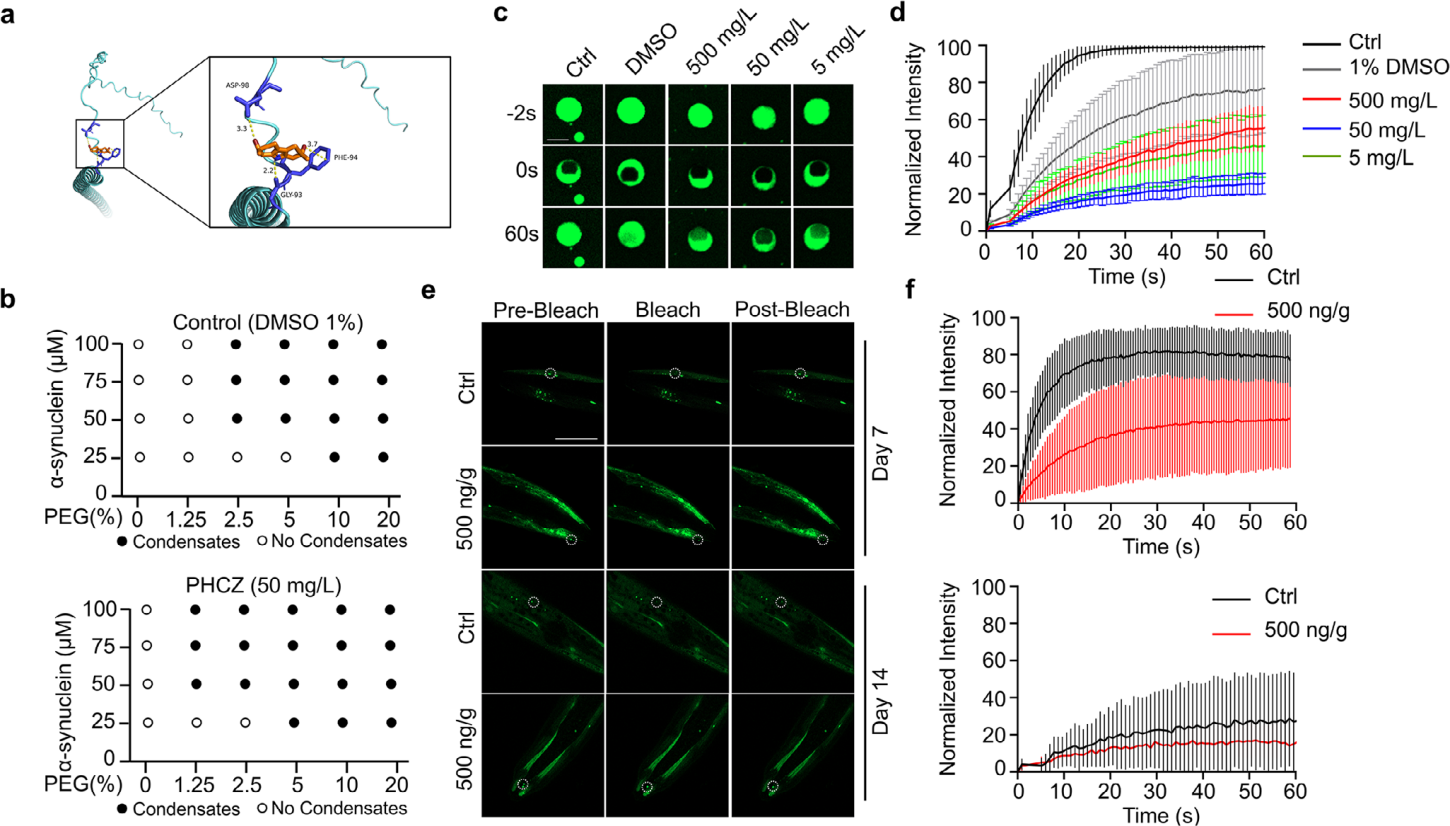
PHCZ promotes condensation of α‐synuclein and reduces its internal fluidity. a) Molecular docking of 2,7‐dibromocarbazole (2,7‐BCZ) with α‐synuclein monomer. b) Phase diagram for different PEG‐8000 concentrations and α‐synuclein concentrations in the absence (Top) and presence of PHCZ (50 mg L^−1^) (Bottom), at which phase separation was observed immediately after incubation. The dots indicate the tested conditions, where hollow dots indicate a lack of phase separation, whilst solid dots indicate phase separation. c) FRAP images of α‐synuclein in vitro. The images correspond to the region of interest with pre‐bleach, bleach, and post‐bleach droplets at ‐2, 0, and 60 s for each tested condition. d) Normalized recovery traces from FRAP experiment for α‐synuclein in vitro (Welch's *t*, two‐sided; BH–FDR within pair; effect size = Hedges’ *g*). 1% DMSO versus Ctrl—MF: *t* (4.15) = −1.11, FDR *p *= 0.328, *g *= −0.63; *t*½: *t* (5.50) = 5.51, FDR *p *= 0.0040, *g *= 3.15; *n *= 5/5. 1% DMSO versus 5 mg L^−1^—MF: *t*(6.47) = 2.42, FDR *p *= 0.0975, *g *= 1.41; *t*½: *t* (8.31) = −0.44, FDR *p *= 0.669, *g *= −0.24; *n *= 5/7. 1% DMSO versus 50 mg L^−1^—MF: *t* (4.61) = 4.50, FDR *p *= 0.0156, *g *= 2.40; *t*½: *t* (6.90) = 0.43, FDR *p *= 0.681, *g *= 0.24; *n *= 5/4. 1% DMSO versus 500 mg L^−1^—MF: *t* (6.91) = 1.12, FDR *p *= 0.298, *g *= 0.64; *t*½: *t* (6.05) = −2.04, FDR *p *= 0.173, *g *= −1.17; *n *= 5/5.). e) FRAP images of α‐synuclein inclusions on day 7 and day 14 of adulthood in NL5901 strains. The images correspond to the region of interest with pre‐bleach, bleach, and post‐bleach droplets at −2, 0, and 40 s for each tested condition. f) Normalized recovery traces from FRAP experiment for α‐synuclein inclusions on day 7 and day 14 of adulthood (top: (Welch's *t*, two‐sided; BH–FDR). t½: *t* (7.35) = −2.82, FDR *p *= 0.0244, *g *= −1.38 (3.62 s vs 13.78 s). *n*: Ctrl = 9, PHCZ = 8. Bottom: *t*½: *t* (11.59) = 1.56, FDR *p *= 0.274, *g *= 0.74 (17.97 s vs 10.37 s). *n*: Ctrl = 8, PHCZ = 9.). Scale bars represent 5 µm in panel (c), and 100 µm in panel (e).

Since α‐synuclein is a key pathological protein in humans with significant biomedical relevance, we quantitatively assessed the binding affinity between PHCZ and α‐synuclein in silico. The predicted Vina score for PHCZ binding to α‐synuclein was ≈−3 (**Figure** **4**a), indicating a moderate binding affinity. Meanwhile, PHCZ treatment markedly lowered the concentration threshold required for recombinant α‐synuclein to form LLPS condensate in vitro (Figure 4b). During the progression of PD, α‐synuclein condensates undergo a transition from fluid‐like to a solid‐like state, which becomes irreversible and ultimately leads to cell death. We next evaluated the internal fluidity of α‐synuclein condensates in vitro using the fluorescence recovery after photobleaching (FRAP) assay. In control samples, the photobleached region recovered rapidly (within 1 min), indicating fluid exchange with other unbleached area within the droplet (Figure 4c,d). However, α‐synuclein condensates pretreated with high concentrations of PHCZ (5 mg L^−1^, ≈10 times of environment relevant concentration) failed to recover from photobleaching, suggesting that PHCZ impairs the internal fluidity of α‐synuclein, promoting its solidification. This PHCZ‐induced solidification of α‐synuclein droplet was further confirmed in vivo using a transgenic *C.elegans* strain expressing α‐synuclein in muscle cells (Figure 4e,f). PHCZ treatment significantly reduced the fluidity of α‐synuclein, supporting the notion that PHCZ facilitates the pathological aggregation of α‐synuclein. Taken together, PHCZ promotes LLPS‐mediated aggregation, contributing to its neurotoxic effects.

### 2.4 Unfolded Protein Response in ER (UPR^ER^) is Induced by PHCZ via *Ire‐1*

**Figure 5:**
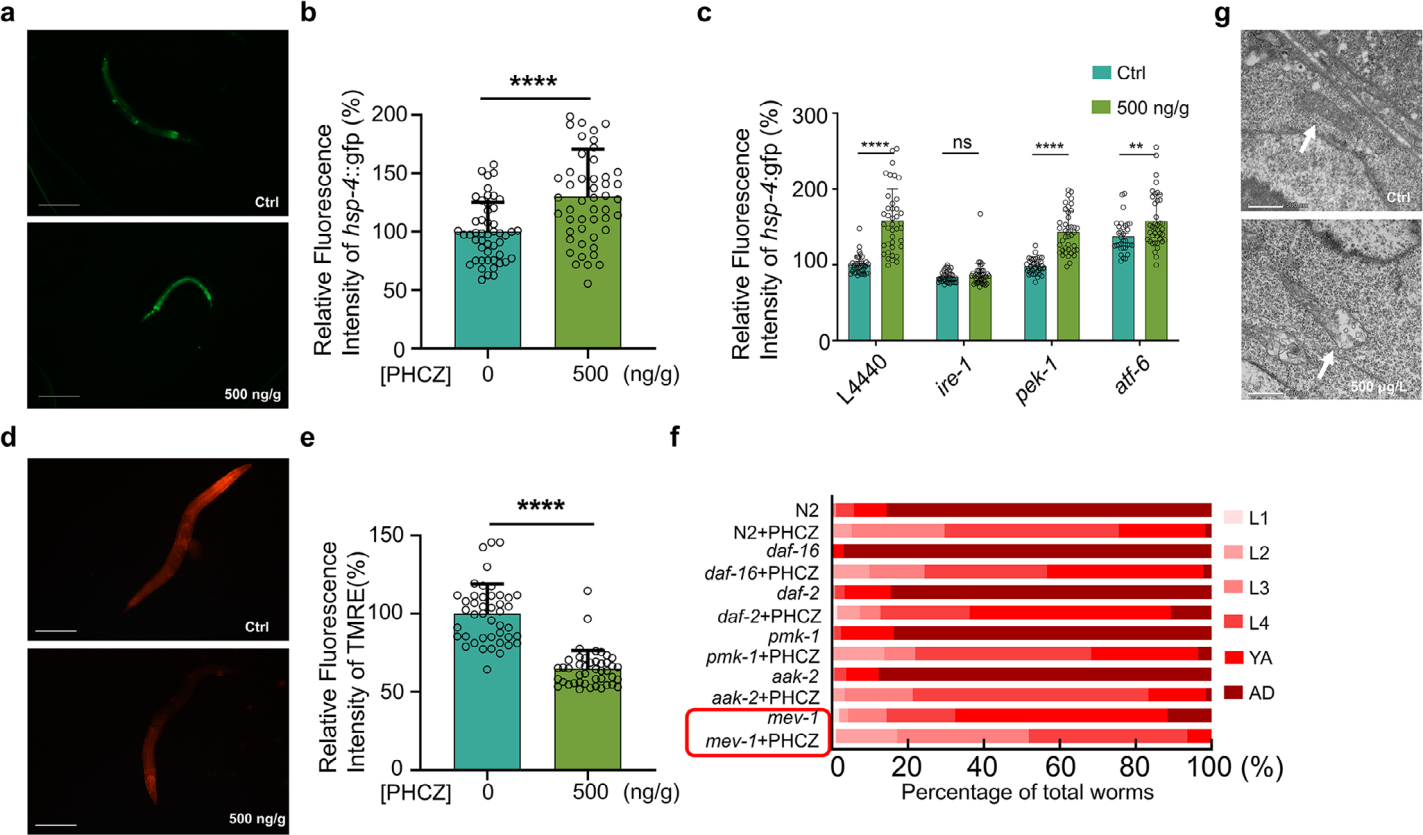
PHCZ compromised mitochondrial integrity and activate *ire‐1* mediated UPR^ER^. a–c) UPR^ER^ activation as indicated by fluorescent intensity of *hsp‐4:gfp* transgenes with indicated treatment and RNAi constructs. Representative images were shown in (a) while their quantifications were shown in (b) (Ctrl (*n *= 49) versus 500 ng g^−1^ (*n *= 49): *t*(96) = −4.38, *p *= 3 × 10^−^⁵; Cohen's *d *= −0.89 (Hedges’ *g *= −0.88).). The representative images of (c) were shown in Figure S4b (Supporting Information; Unpaired *t* test (FDR‐corrected, two‐tailed): L4440 *t* (78) = 8.51, *p *< 0.000001, *g *= 1.90; *ire‐1 t*(78) = 0.96, *p *= 0.341, *g *= 0.21; *pek‐1 t*(79) = 9.77, *p *< 0.000001, *g *= 2.19; *atf‐6 t*(70) = 2.83, *p *= 0.0061, *g *= 0.67; *n *= 35–41/group.). d,e) Mitochondrial membrane potential was dampened with 500 ng g^−1^ PHCZ treatment. The membrane potential was measured with TMRE staining, and its quantification was shown in e (Unpaired Student's *t* (two‐sided). Control (*n *= 44) versus 500 ng g^−1^ (*n *= 44): *t* (86) = 10.49, *p *= 4.77 × 10^−1^ ^⁷^, Hedges’ *g *= 2.22.). f) PHCZ synergizes with *mev‐1* deficiency in delaying the development of *C. elegans*. The percentage of worms with indicated genotypes and treatment at each indicated stage were quantitated. ((L1–AD; *χ* ^2^ 2 × *k*, BH–FDR; Cramér's *V*). N2 versus PHCZ+N2: *χ* ^2^(5) = 146.99, FDR *p *= 1.17 × 10^−2^⁹, *V* = 0.86; *aak‐2* versus PHCZ+*aak‐2*: *χ* ^2^(4) = 154.56, *p *= 6.43 × 10^−3^ ^2^, *V* = 0.88; *daf‐16* versus PHCZ+*daf‐16*: *χ* ^2^(4) = 162.89, *p *= 2.1 × 10^−3^, *V* = 0.89; *daf‐2* versus PHCZ+*daf‐2*: *χ* ^2^(5) = 110.57, *p *= 3.73 × 10^−2^, *V* = 0.74; *mev‐1* versus PHCZ+*mev‐1*: *χ* ^2^(5) = 84.25, *p *= 1.08 × 10^−2^⁶, *V* = 0.65; *pmk‐1* versus PHCZ+*pmk‐1*: *χ* ^2^(4) = 122.42, *p *= 2.43 × 10^−2^⁵, *V* = 0.77.) g) Representative EM images of mitochondria from worms with the indicated treatment. Scale bars represent 50 µm in panel (a,d) and 0.5 µm in panel (g). Data were shown as mean ± SEM in (b,c,e). ns: not significantly different; *: *p *< 0.05; **: *p *< 0.01; ***: *p *< 0.001; ****: *p *< 0.0001.

Given the capacity of PHCZ to interrupt LLPS and the fact that many proteins across different cellular compartments are structurally organized through the LLPS mechanism, we investigated whether PHCZ induces protein homeostasis stress in cells. To address this, we utilized three reporter strains to monitor the unfolded protein responses (UPR) in the cytosol, ER, and mitochondria respectively. The data revealed that UPR^ER^ (*hsp‐4*::GFP, **Figure** **5**a,b) was specifically activated, whereas UPR^mito^ (*hsp‐6*::GFP and *dve‐1*::GFP, Figure S4a–c, Supporting Information) and cytosolic UPR (*hsp‐16.2*::GFP, Figure S4d,e, Supporting Information) were not, indicating that PHCZ may act as a general disruptor of ER homeostasis. UPR^ER^ can be activated through three parallel pathways: IRE‐1, PEK‐1, and ATF‐6 (Figure S5a, Supporting Information). Epistatic analysis showed that PHCZ‐induced UPR^ER^ depends on the *ire‐1* pathway, while the other two pathways are not involved (Figure 5c; Figure S5b, Supporting Information). Consistent with the role of UPR^ER^ in resolving the protein homeostasis crisis,^[^ ^50^ ^]^ we found that RNAi of *ire‐1* significantly upregulated the expression level of α‐synuclein::EGFP and also tantalizingly worsened its condensation in day 3 adult worms (Figure S5c,d, Supporting Information). This suggests that *ire‐1*‐mediated UPR^ER^ prevents abnormal condensation of α‐synuclein. While sham RNAi worms exposed to PHCZ exhibited more α‐synuclein::EGFP condensates than unexposed worms at day 3 of adulthood, PHCZ did not further exacerbate the condensation phenotype in *ire‐1* RNAi‐treated worms. The lack of an additive effect between PHCZ and *ire‐1* RNAi likely supports the idea that PHCZ promotes pathological α‐synuclein condensation by inhibiting the *ire‐1*‐mediated UPR^ER^ pathway. To address the physiological difference between worms and humans, we titrated PHCZ in SH‐SY5Y cells and found that 50 µm PHCZ robustly induced IRE1α phosphorylation, indicating activation of the IRE1α‐dependent UPR^ER^ in human cells (Figure S5e, Supporting Information).

### 2.5 Mitochondrial Integrity was compromised in PHCZ‐exposed worms and Human Cells

Mitochondrial malfunction has been shown as one primary driving force for PD. Given this, we investigated whether PHCZ exposure impairs mitochondrial function. First, we observed that worms treated with 500 ng g^−1^ PHCZ exhibited a marked decrease of mitochondrial membrane potential (MMP) compared to control worms (Figure 5d,e). We then assessed the protein expression and functionality of the mitochondrial electron transport chain (ETC) complexes, while mtDNA abundance as normalized to nucleus DNA remains unchanged (Figure S6a,b, Supporting Information). Notably, the protein levels of key enzymes in ETC complexes (I–V) exhibited differential alterations (Figure S6a, Supporting Information). However, no ROS production, which is sometimes associated with ETC dysfunction, was observed in worms treated with PHCZ (Figure S6c,d, Supporting Information). We hypothesized that the decrease in MMP underpins PHCZ‐induced toxicity. To explore this, we examined the development trajectory of *mev‐1* mutants, which carry a mutation in a nuclear‐encoded ETC complex II enzyme. Interestingly, we found that *mev‐1* mutants had a delayed development timeline, potentially recapitulating the PHCZ toxicity (Figure 5f). However, when PHCZ treatment was combined with *mev‐1* deficiency, a substantial additive effect was observed, suggesting that PHCZ and *mev‐1* operate through parallel pathways to maintain normal developmental physiology in worms. Given that ETC complex I and II fuel electrons to complex III by oxidizing NADH (complex I) and FADH_2_ (complex II), it is plausible that PHCZ specifically targets complex I, which could synergize with *mev‐1* deficiency in disrupting electron transport. *mev‐1* deficiency extends longevity in *C. elegans*, we thus examined other pathways associated with longevity and stress responses, and found that *pmk‐1* (innate immunity), *daf‐2/daf‐16* (insulin‐like signaling), and *aak‐2* (AMPK) did not influence the developmental disturbance caused by PHCZ in *C. elegans* (Figure 5f). Consistent with the mitochondrial involvement in PHCZ toxicity, high‐resolution electron microscopy revealed that PHCZ caused significant destruction of mitochondrial structures in the somatic tissues of *C. elegans* (Figure 5g).

We demonstrated that PHCZ did not affect mitochondrial function via oxidative stress, as antioxidant treatment failed to rescue PHCZ‐mediated mitochondrial membrane potential loss (Figure S7a,b, Supporting Information) or dopaminergic neuron loss in *C.elegans* (Figure S7c, Supporting Information). In SH‐SY5Y and HeLa cells, we evaluated PHCZ‐induced mitochondrial toxicity. PHCZ reduced mitochondrial membrane potential (Figure S8a, Supporting Information) and activated the PINK1‐Parkin mitochondrial quality‐control pathway (Figure S8b–e, Supporting Information). Specifically, in HeLa cells, 100 µm PHCZ triggered Parkin translocation from cytosol to mitochondria and increased PINK1‐mediated Parkin phosphorylation, indicating mitochondrial dysfunction (Figure S8c, Supporting Information). Consistently, PHCZ elevated phosphorylation of polyubiquitinated proteins, a marker of PINK1‐Parkin pathway activation, in both SH‐SY5Y and HeLa cells (Figure S8d,e, Supporting Information). These findings suggest that compromised mitochondrial function is a key molecular event underlying PHCZ toxicity.

## 3 Discussion

PHCZ is an emerging organic contaminant with widespread occurrence, primarily released through industrial emissions from textile printing and dyeing processes.^[^ ^5^ ^]^ Despite its prevalence, the potential health threats and ecological risks associated with PHCZ remain largely unexplored. The canonical perspective attributed the toxicity of PHCZ to the activation of AHR due to structural similarity with dioxins.^[^ ^1^ ^]^ However, we demonstrated that PHCZ exposure at environmentally relevant concentrations results in general developmental toxicity independent of AHR. We unexpectedly found that PHCZ treatment at 5 ng g^−1^ caused prolonged menopause in *C. elegans*. This dose‐dependent pattern aligns with hormesis.^[^ ^51^ ^]^ Reproductive period extension occurred only at low doses but not higher doses, while brood size defects were associated with medium and high doses treatment but not low doses. The observed reproductive toxicity is consistent with a previous study that reported PHCZ promiscuously interacts with estrogen receptor and resulted in aberrant changes in uterine epithelium cell heights and relative uterus weights.^[^ ^13^ ^]^

PHCZ can induce dopaminergic neuron degeneration in *C. elegans*. Mechanistically, our findings revealed that PHCZ dysregulates the LLPS of α‐synuclein by reducing the fluidity of its condensate. This suggests that PHCZ may promote neuronal cell death by converting the dynamic LLPS condensates into a more solid‐like state. LLPS plays a crucial role in organizing and regulating various cellular processes, including transcription,^[^ ^52^ ^]^ translation,^[^ ^53^ ^]^ small RNA‐mediated gene silencing,^[^ ^54^ ^]^ and the pathological progression of neurodegenerative diseases.^[^ ^55^ ^]^ Given the centrality of LLPS in cellular function, it may represent a common target for organic pollutants. Various charged nanoparticles, such as graphene nanoparticles (<100 nm in diameter), silica, zirconium, and polyethylenimine‐coated carboxyl‐modified polystyrene nanoparticles, have been shown to influence α‐synuclein fibril nucleation and elongation^[^ ^4^, ^56^, ^57^, ^58^ ^]^ in vitro. Additionally, anion nanoplastic particles have been found to catalyze the formation and propagation of α‐synuclein fibrils in vivo due to their unique surface property. Sun *et al*. further demonstrated that polystyrene nanoparticles induced aberrant condensation of another phase‐separable pathological protein, TDP‐43, promoting amyotrophic lateral sclerosis‐like symptoms.^[^ ^19^ ^]^ While nanoplastics, with their high surface free energy, are known to interfere with LLPS, our study raised the question: are other contaminants capable of disrupting LLPS with a distinct mechanism? Notably, *C. elegans* lacking ectopic expression of human α‐synuclein still exhibited dopaminergic neuron loss upon exposure to PHCZ, indicating that PHCZ may interfere with the condensation of the worm counterpart of α‐synuclein or functional analogs. Unlike nanoplastic particles, which are internalized in cells primarily via clathrin‐dependent endocytosis and routed to lysosomes, PHCZ, as an organic compound, may directly cross the plasma membrane and accumulate in the cytosol, where α‐synuclein normally resides.^[^ ^59^ ^]^ This direct access to the cytosol suggests that PHCZ could exert more detrimental effects than solid particles.

Interestingly, as an indicator of protein homeostasis stress, UPR particularly in the ER was activated by PHCZ. PHCZ may have broad reactivities due to its aromatic ring and halogen atoms, allowing it to promiscuously bind yet identified proteins. Additionally, we observed that PHCZ severely compromised mitochondrial membrane potential. Given the pivotal role of mitochondria in neuronal function, including neuroregeneration and axon repair,^[^ ^60^, ^61^ ^]^ this mitochondrial disruption may further contribute to the neurotoxicity of PHCZ. Compared to PHCZ, nanoplastics also activate common stress pathways, including ER stress/UPR^ER^ and mitochondrial malfunction, but typically via indirect mechanisms such as ROS elevation, Ca^2^⁺ dysregulation, and lysosomal injury. These pathways converge on UPR^ER^ markers and mitochondrial depolarization across models.^[^ ^62^, ^63^ ^]^

To situate these mechanistic observations in a mammalian context, we intersected predicted PHCZ targets with Parkinson's disease gene sets and analyzed the overlap (Figure S9a, Supporting Information). GO/KEGG enrichment of the intersecting genes highlighted neurodegeneration‐relevant pathways (Figure S9b,c, Supporting Information), while protein–protein‐interaction (PPI) mapping ranked α‐synuclein (SNCA) among the top hubs and resolved modules annotated for dopaminergic synapse and proteostasis (Figure S9d, Supporting Information). This in silico signature aligns with our experimental observations that convergence on an α‐synuclein‐centered proteostasis axis.

Overall, PHCZ, as an organic chemical that dissemble the nanoparticle contaminates, can also target LLPS, which may suggest LLPS dysregulation could be a general mechanism of PHCZ and many other uncharacterized toxicants. Furthermore, protein homeostasis dysregulation may also represent a common toxic target for PHCZ and potentially other uncharacterized toxicants, highlighting the need for deeper investigation into their broad toxicological impacts.

## 4 Experimental Section

### *C. elega*ns Strain

N2 (wildtype), NL5901 (*pkIs2386*[*unc‐54p*::α‐synuclein::YFP]), CL2166 (*dvIs19*[*pAF15*(*gst‐4*::GFP::NLS)]), DA1240 (*adIs1240*[*eat‐4*::sGFP + lin‐15(+)]), BZ555 (*egIs1* [*dat‐1p::GFP*]), UA44 (*baIn11*[*dat‐1p::GFP; dat‐1p::α‐synuclein*]), GR1333 (*yzIs71*[*tph‐1p::GFP* + *rol‐6(su1006)*]), *zcIs4* [*hsp‐4*::*GFP*], *ahr‐1*(*ju145)*, *him‐5(e1467)*, *pmk‐1(km25), daf‐16(mu86), daf‐2(e1368), aak‐2(ok524), and mev‐1(kn1)*.

### Maintenance and Exposure of *C. elegans*

All *C. elegans* strains were maintained on Nematode Growth Medium (NGM) plates seeded with *Escherichia coli* (*E. coli)* OP50 at 20 °C. For the exposure experiments, synchronized L1 was cultured on NGM plates containing 2,7‐BCZ for 72 h. Synchronization was done by bleaching pregnant hermaphrodite in bleach solution (0.45 m NaOH and 2% HOCl), and the obtained eggs were transferred to NGM plates.

### Lifespan

The synchronized L4 animals were transferred to NGM plates containing 75 µm 5‐Fluorouracil and 2,7‐BCZ and maintained at 20 °C. Survival was monitored every day, and worms that failed to respond to gentle touch were considered dead. Statistical analyses were performed after all worms were dead. Each group consisted of at least 60 worms.

### Locomotion Behavior

After exposure to 2,7‐BCZ, the animals were placed on OP50‐free NGM plates and allowed to recover for 1 min. The number of head thrashes within 20s was recorded. At least 40 worms were assessed for each experimental group.

### Developmental Staging

*C. elegans* were classified into stages based on size and developmental characteristics: L1, L2, L3, L4, young adult (YA), and adult (AD). The number of *C. elegans* at each stage was counted on plates, with each worm euthanized by heating to prevent recounting. At least 50 worms were analyzed per group.

### Brood Size

To assess the brood size of hermaphrodites, a single synchronized L4 hermaphrodite was transferred to an individual NGM plate and transferred every 24h until egg‐laying ceased, and the number of progenies produced was counted at the L4 stage. The total brood size was determined by counting the total number of offspring.

### Reproductive Span

Synchronized *C. elegans* N2 were exposed to PHCZ and transferred daily to fresh NGM plates. Each worm was placed on a separate plate to avoid cross‐contamination of offspring between worms. The worms were monitored for the production of offspring each day, and the transfer continued until no further offspring were produced. The reproductive span was defined as the number of days between the first and last egg‐laying events. The data were used to calculate the reproductive span for each individual worm.

### Sperm Morphology

Synchronized male worms were transferred to fresh plates after 48h of exposure for an additional 24 h, which allowed them to accumulate sperm without mating with hermaphrodites. The exposed males were then transferred to OP50‐free NGM plates for 30 min to remove *E. coli*. A drop of Sperm Medium Buffer (SM Buffer, 50 mm HEPES, 1 mm MgSO_4_, 25 mm KCl, 45 mm NaCl, 5 mm CaCl_2_, and pH 7.0–7.8) was placed on a glass slide, and males were placed in the buffer. Worms were dissected at three‐quarters of the body to release sperm. Sperm morphology was observed under a microscope, and the number of abnormally shaped sperm was recorded, with at least 200 sperm counted per group.

### Metabolism Analysis

Synchronized adult nematodes were harvested, washed with M9 buffer, and flash‐frozen in liquid nitrogen. The metabolism sequencing and data analysis were performed by Metware (Wuhan, China). Unsupervised PCA (principal component analysis) was performed by the statistics function prcomp within R. The data was unit variance scaled before unsupervised PCA.

### RNA‐Seq Experiment and Data Analysis

Synchronized adult nematodes were harvested, washed with M9, and flash‐frozen in liquid nitrogen. The RNA sequencing was performed by BGI Genomics (Wuhan, China). Clean reads were aligned to the *C. elegans* reference genome (WBcel235).

Differential gene expression analysis was conducted using Rstudio, applying a false discovery rate (FDR) < 0.05 and a |log2 fold‐change| > 1 to identify significantly differentially expressed genes. Gene ontology (GO) enrichment analysis and Kyoto Encyclopedia of Genes and Genomes (KEGG) pathway analysis were carried out using Metascape and DAVID.

### RNAi

*C. elegans* were fed with *E. coli* HT115 containing an empty vector L4440 or *E. coli* from the RNAi library expressing *ahr‐1*, *ire‐1*, *pek‐1*, and *atf‐6* double‐stranded RNA.

### Sperm Activation In Vitro

Males were picked after 48 h exposure for another 24 h exposure. After 24 h, males were allowed to crawl on the NGM plate without food for 30 min. At least 10 worms were dissected in SM Buffer containing 200 mg L^−1^ Pronase E, the proportion of activated sperm was counted after 15 min.

### Neurodegeneration Analysis

To assess dopaminergic neuron degeneration, head neurons of BZ555 worms were categorized as normal condition, morphological deviation, blebs, or breaks. The percentage of worms exhibiting abnormalities was recorded. For serotonergic neurons, GR1333 worms were used to count the number of NSM neurons.

### α‐Synuclein Aggregation and Neurodegeneration Analysis in *C. elegans*

Synchronized NL5901, UA44 worms were transferred to plates containing 500 ng g^−1^ 2,7‐BCZ on the first day of adulthood and exposed for 7 days. Worms were then imaged under a fluorescence microscope, with at least 40 worms analyzed per group. For UA44 worms, the proportion of intact CEP neurons was recorded. NL5901 worms were classified into three types based on the degree of α‐synuclein aggregation: Normal condition, Moderate aggregation, and Advanced aggregation, Normal: 0–5 discernible α‐syn droplets in the head region; Moderate aggregation: 6–15 α‐syn droplets; Advanced aggregation: more than 16 α‐syn droplets. The proportions of these three types of worms were quantified. In antioxidant intervention experiments, 5 mm vitamin C (VC), 1 mm N‐acetylcysteine (NAC), or 50 µm lycopene was added to the plates together.

### In Vivo FRAP Analysis of a‐Synuclein Condensates

NL5901 worms were transferred to agarose pads on glass slides and anesthetized with sodium azide. FRAP was performed using a Zeiss LSM 900 confocal microscope with a 63× oil immersion objective (Zeiss, Germany). The excitation wavelength was set to 488 nm. A region for photobleaching was selected, with 100% laser power used for bleaching. The intensity of the bleached region was background‐corrected and normalized with a reference signal and FRAP time.

### α‐Synuclein Purification

The plasmids pET28a‐SUMO‐α‐syn and pET28a‐SUMO‐α‐syn‐EGFP were kindly provided by Dr. Haijia Yu. α‐syn and α‐syn‐EGFP were purified following the protocol of Xu *et al*., with modifications.^[^ ^64^ ^]^ Briefly, the plasmids were transformed into *E. coli* BL21, cultured in flasks at 37 °C until OD600 reached 0.6–0.8, and induced with IPTG at a final concentration of 1 mm for 12 h at 18 °C. Bacterial cells were collected by centrifugation at 8000 rpm at 4 °C, resuspended in PBS, and lysed by sonication for 30 min. After centrifugation at 8000 rpm, the supernatant containing His6‐SUMO‐α‐syn and His6‐SUMO‐α‐syn‐EGFP fusion proteins was purified using nickel affinity chromatography. The SUMO tag was removed by cleavage with SUMO protease. Unlabeled protein was dialyzed overnight in storage buffer (25 mm Tris‐HCl [pH 7.4], 50 mm NaCl) and stored at −80 °C after lyophilization.

### In Vitro FRAP Analysis of α‐Synuclein

Purified α‐syn and α‐syn‐EGFP were mixed at a molar ratio of 9:1.^[^ ^64^ ^]^ A mixture containing 200 µm α‐syn and 20% PEG‐8000 was prepared to form liquid droplets. A 10 µL droplet was placed on a slide and covered with a coverslip. FRAP was performed on a Zeiss LSM 900 confocal microscope with a 63× oil immersion objective (Zeiss, Germany) at an excitation wavelength of 488 nm. A region for photobleaching was selected with 100% laser power. The intensity of the bleached region was background‐corrected and normalized using a reference signal and FRAP time.

### Molecular Docking

The 2D structure of 2,7‐BCZ was downloaded from PubChem, and the crystal structures of α‐syn were obtained from the Protein Data Bank (PDB). Molecular docking and visualization were performed using AutoDock Vina, with binding energy calculated.

### Mitochondrial Membrane Potential Measurement

TMRE dye (Beyotime) was diluted 100‐fold in M9 buffer and added to NGM plates for overnight staining, shielded from light. In antioxidant intervention experiments, 5 mm vitamin C, 1 mm N‐acetylcysteine, or 50 µm lycopene was added to the plates. After staining, worms were anesthetized and imaged under a fluorescence microscope. Fluorescence intensity was analyzed using ImageJ software, with at least 40 worms examined per group.

### Cell Culture and Exposure

HEK293T cells were cultured in DMEM supplemented with 10% fetal bovine serum and 1% antibiotics. Cells were seeded in 6‐well plates at a density of 10^6^ cells per well. After 12h of attachment, the medium was replaced with fresh medium containing 2,7‐BCZ, and the medium was refreshed every 24h. Cells were collected after 72 h of exposure.

### Western Blot Analysis

HEK293T, Venus–parkin HeLa, and SH‐SY5Y cells were exposed to PHCZ 24 h, then collected and lysed in lysis buffer. Lysates were centrifuged at 12,000 rpm at 4 °C for 10 min, and supernatants were mixed with loading buffer and boiled for 10 min. Equal amounts of protein were loaded and probed with the following primary antibodies: Total OXPHOS human antibody cocktail (Abcam, UK, AB110413) for HEK293T cells; anti‐parkin (CST, USA, 4211T) and anti‐phospho‐ubiquitin (CST, USA, 62802T) for Venus–parkin HeLa cells; anti‐phospho‐ubiquitin and anti‐phospho‐IRE1α (Abcam, UK, AB48187) for SH‐SY5Y cells.

### Quantification of mtDNA/Nuclear DNA Ratio by qPCR

Total genomic DNA was extracted from *C. elegans* using DNA extract kit (TIANGEN, China). qPCR was performed with primers targeting mitochondrial *tRNA‐Leu* (tRNA‐Leu‐F: CACCCAAGAACAGGGTTTGT/tRNA‐Leu‐R: TGGCCATGGGTATGTTGTTA) and nuclear *B2G* (B2G‐F: TGCTGTCTCCATGTTTGATGTATCT/B2G‐R: TCTCTGCTCCCCACCTCTAAGT). Reactions included 2× SYBR Green Master Mix, primers (0.5 µm each), and 50 ng DNA in a 20 µL volume. The mtDNA/nDNA ratio was calculated using the ΔCt method as 2^−ΔΔCt^.

### Transmission Electron Microscopy (TEM)

Large numbers of *C. elegans* were collected, washed in M9 buffer, and immediately placed in 2.5% glutaraldehyde fixative (Baiqiandu Biotechnology, Wuhan) at 4 °C for fixation and storage. Images were subsequently acquired.

### UPR^ER^, UPR^mito^, and Oxidative Stress Test

*hsp‐4*::*GFP*, *hsp‐6*::*GFP*, *dve‐1p::dve‐1::GFP*, *hsp‐16.2::GFP*, and CL2166 worms were used to detect endoplasmic reticulum stress, mitochondrial stress, cytosolic stress, and oxidative stress, respectively. Synchronized *hsp‐4*::*GFP*, *hsp‐6*::*GFP*, *dve‐1p::dve‐1::GFP*, *hsp‐16.2::GFP*, and CL2166 worms were placed on NGM plates containing 500 ng g^−1^ 2,7‐BCZ for 72 h until 1 day of adulthood. At least 40 worms from each treatment group were imaged, and fluorescence intensities were quantified by ImageJ.

### JC‐1 Staining

SH‐SY5Y cells were exposed to PHCZ for 24 h, then incubated with JC‐1 working solution (Beyotime) at 37 °C for 20 min in the dark. After washing twice with PBS, fluorescence was observed using a fluorescence microscope (red, aggregated JC‐1; green, monomeric JC‐1)

### Confocal Imaging

Venus–parkin HeLa cells were exposed to PHCZ for 24 h, then incubated with MitoTracker Red (200 nm, Beyotime) at 37 °C for 30 min, followed by staining with DAPI 10 µg mL^−1^, Beyotime) for 5 min. After washing twice with pre‐warmed PBS, live‐cell imaging was performed using a confocal microscope (AX, Nikon) with appropriate excitation/emission settings for Venus, MitoTracker Red, and DAPI.

SH‐SY5Y cells were exposed to PHCZ for 24 h, fixed with 4% paraformaldehyde for 15 min, permeabilized with 0.1% Triton X‐100 for 10 min, and blocked with 5% BSA for 1 h. Cells were then incubated with anti‐Ki67 antibody (CST, USA, 4 °C overnight), followed by incubation with an Alexa Fluor‐conjugated secondary antibody for 1 h at room temperature. After DAPI counterstaining, images were acquired using the same confocal system. The percentage of Ki67‐positive cells was quantified from at least five randomly selected fields using ImageJ.

For Calcein‐AM staining, SH‐SY5Y cells were exposed to PHCZ for 24 h, washed with PBS, and incubated with calcein‐AM (5 µm, Beyotime) at 37 °C for 30 min in the dark. After washing twice with PBS, live‐cell imaging was performed using the same confocal microscope with appropriate settings for calcein fluorescence.

### Network Toxicology Analysis

PHCZ targets were predicted from PubChem canonical SMILES using SuperPred, NetInfer, and SEA; weak‐affinity interactions (Ki/IC50 > 10 µm) were removed, and targets were merged and deduplicated at the gene level. Parkinson's disease genes were retrieved from GeneCards (query “parkinson,” *Disorders* → Protein Coding) and OMIM and deduplicated. The intersecting genes underwent GO and KEGG enrichment with clusterProfiler (Benjamini–Hochberg adjustment; FDR < 0.05; top five GO terms visualized) and PPI retrieval via STRING (*Homo sapiens*, default), followed by Cytoscape v3.10.3 visualization; modules were identified with MCODE and summarized by their top three KEGG pathways (by gene count).

## Conflict of Interest

The authors declare no conflict of interest.

## Supporting information



Supporting Information

Supporting Information

## Data Availability

The data that support the findings of this study are available from the corresponding author upon reasonable request.
