# Supplementary material for: Polyhalogenated Carbazole Impairs Dopaminergic Neurons through Dysregulation of Liquid–Liquid Phase Separation in Caenorhabditis elegans
Source: Adv Sci (Weinh). 2025 Oct 9;12(48):e02486. doi: 10.1002/advs.202502486 (PMC12752670; doi:10.1002/advs.202502486)
Supplement: Supplementary file 1 — Supporting Information [file ADVS-12-e02486-s002.docx]

**Supplemental Information:**


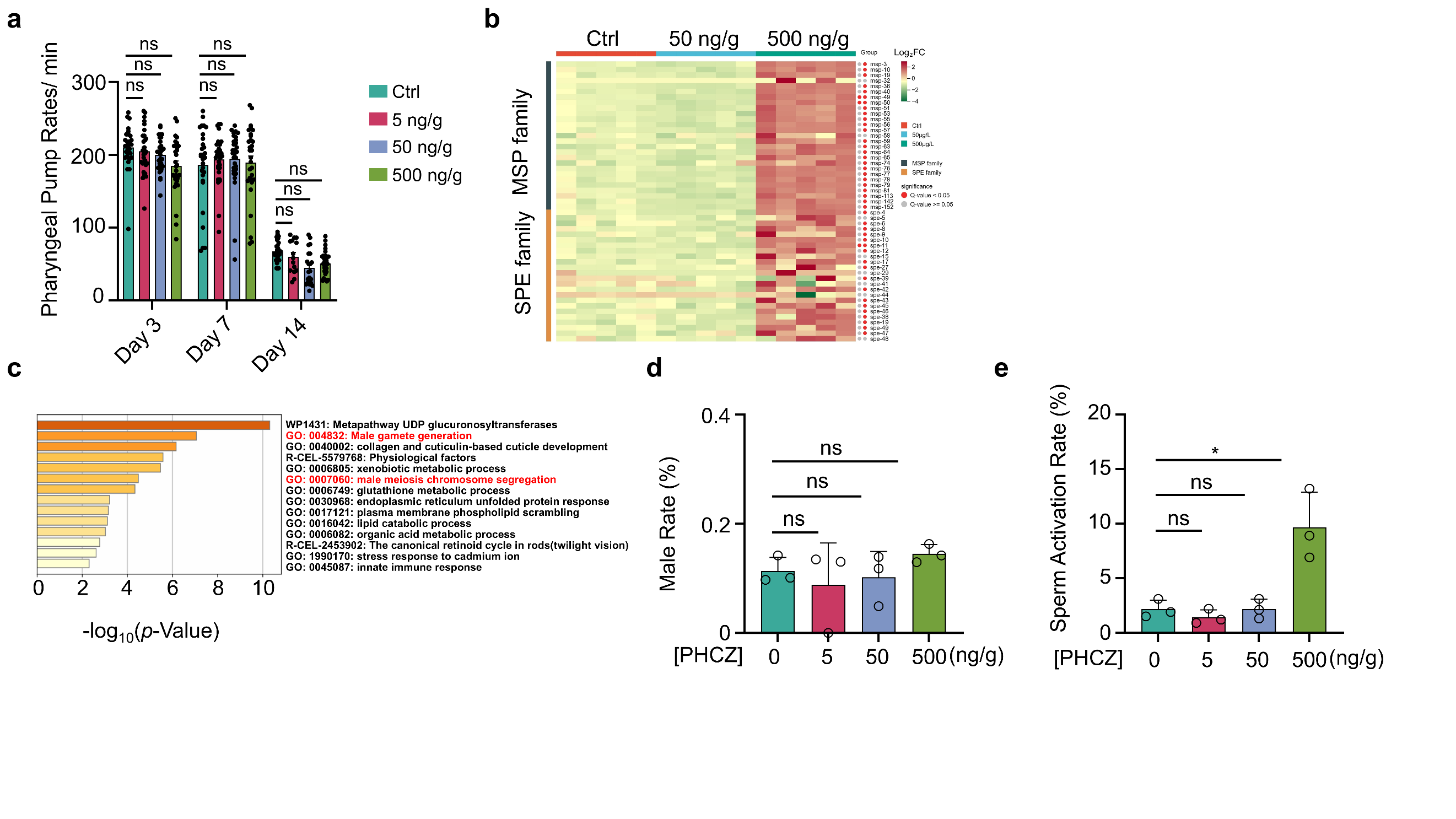


**Figure S1. Sperm development was severely impaired by PHCZ treatment.** a) Pharyngeal pump rates *per* min of worms with indicated treatment. (Šídák’s post hoc vs. Ctrl; within-day adjustment.

Day 3: 5 ng/g: Δ = 5.27 [−17.95, 28.49], adjust *p* = 0.992; 50 ng/g: Δ = 10.34 [−12.88, 33.56], adjust *p* = 0.806; 500 ng/g: Δ = 22.07 [−1.15, 45.29], adjust *p* = 0.072 (n = 29/30 each).Day 7: 5 ng/g: Δ = −7.53 [−30.75, 15.69], adjust *p* = 0.949; 50 ng/g: Δ = −8.47 [−31.69, 14.75], adjust *p* = 0.914; 500 ng/g: Δ = −3.33 [−26.55, 19.89], adjust *p* = 0.999 (n = 29/30 each).Day 14: 5 ng/g: Δ = 7.41 [−21.50, 36.33], adjust *p* = 0.984 (n = 26/15); 50 ng/g: Δ = 22.62 [−3.21, 48.45], adjust *p* = 0.120 (n = 26/22); 500 ng/g: Δ = 16.81 [−7.08, 40.71], adjust *p* = 0.325 (n = 26/30).) b) Heatmap of SPE family genes that play important roles in sperm differentiation and normal function. c) GO Pathway enrichment revealed multiple pathways that include the male gamete generation and male meiosis chromosome segregation. d) Male rates of worms with indicated treatments. (Two-sided Welch’s *t* with Holm correction across three contrasts; effect size = Hedges’ *g*; *n*=3 *per* group. 5 ng/g: *t* (2.41) =0.54, Holm *p*=1.00, *g*=0.36; 50 ng/g: *t* (3.02) =0.37, Holm *p*=1.00, *g*=0.24; 500 ng/g: *t* (3.53) =−1.82, Holm *p*=0.457, *g*=−1.19.) e) Sperm activation rates of worms with indicated treatments. (One-way ANOVA with Dunnett’s post hoc test (*vs.* 0 ng/g); values are mean difference Δ (95% CI), adjusted *p*; *n*=3/group. 0 ng/g *vs.* 5 ng/g: Δ=1.19 [−15.09, 17.47], adjusted *p* =0.993; 0 ng/g *vs.* 50 ng/g: Δ=11.82 [−4.46, 28.10], adjusted *p* =0.161; 0 ng/g *vs.* 500 ng/g: Δ=20.48 [4.20, 36.76], *p adj* =0.017.) Data were shown as mean ± SEM in a), d), and e). ns: not significantly different; *: *p*<0.05; **: *p*<0.01; ***: *p*<0.001; ****: *p*<0.0001.


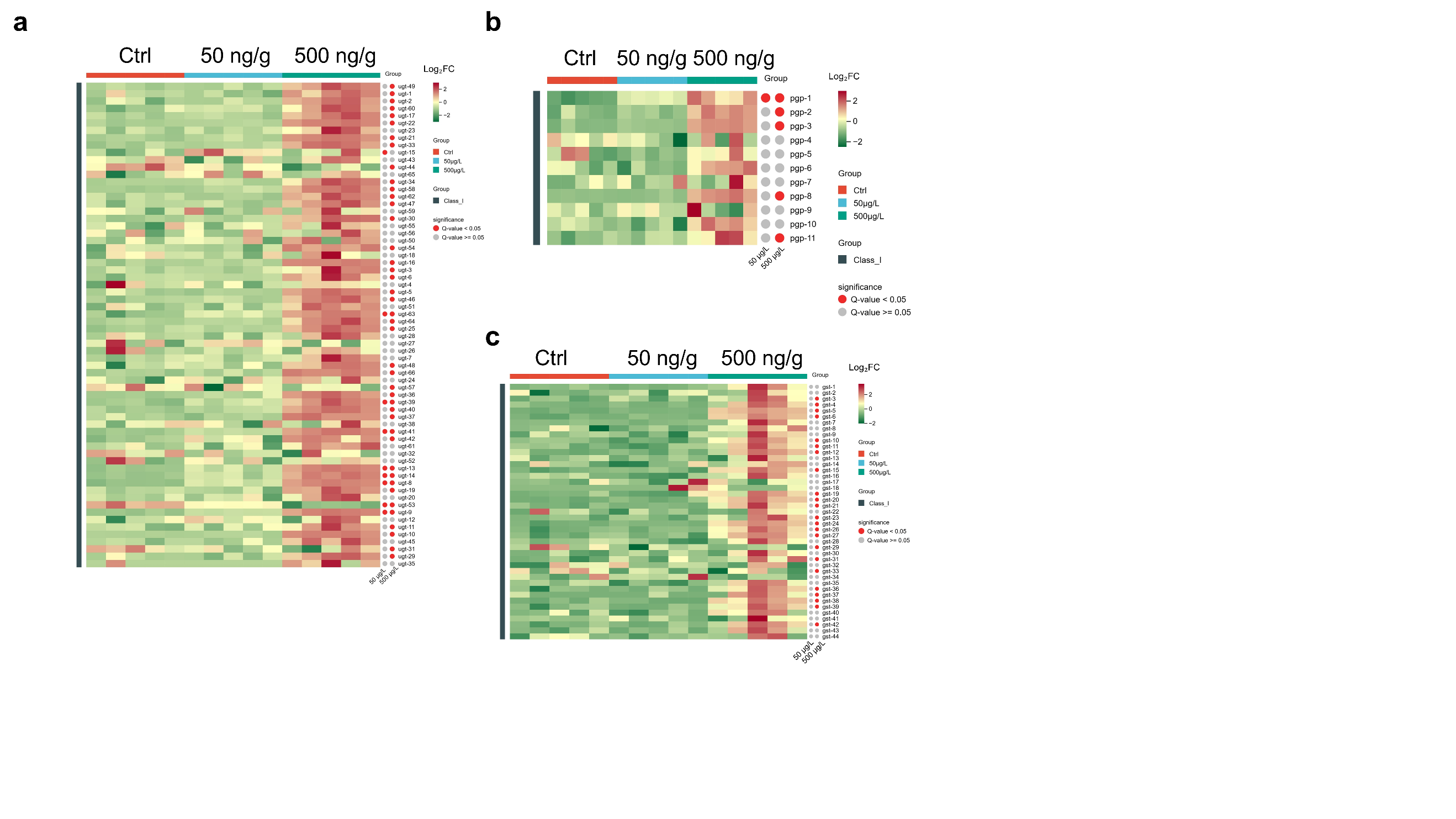


**Figure S2. Heatmaps of other detoxifying genes.** Heatmaps of a) *ugt* family gene, b) *pgp* family gene, and c) *gst* family genes.


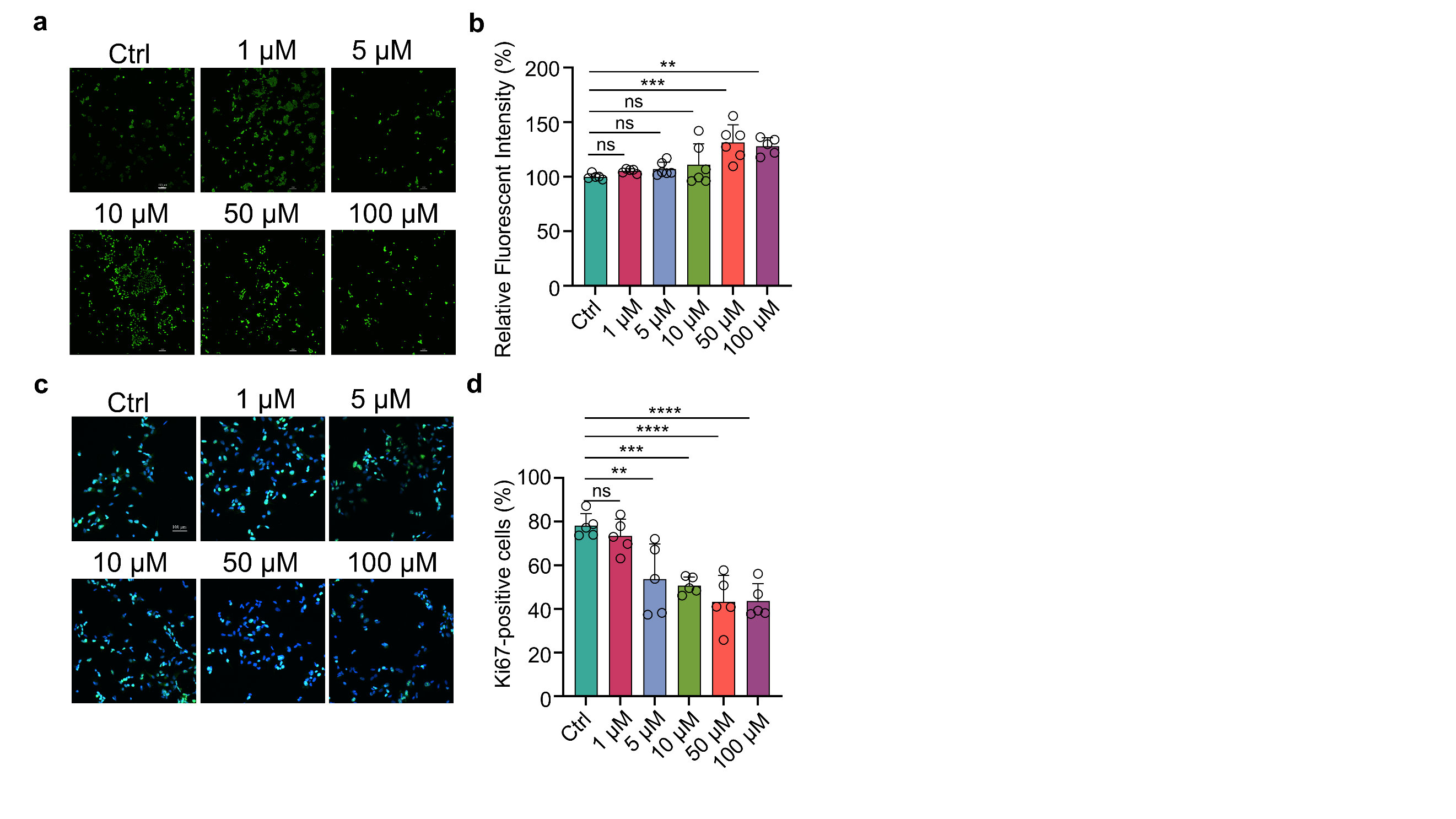


**Figure S3. Calcein-AM staining and cell proliferation of SH-SY5Y cells after being exposed to PHCZ.** a) Representative images of SH-SY5Y cells stained with Calcein-AM following PHCZ treatment. The Calcein-AM fluorescence indicated the membrane permeability by monitoring the activities of ABC transporter dependent exflux. b) Quantification of relative fluorescence intensity of Calcein-AM. (One-way ANOVA: F (5,27) =6.76, *p*=0.0003, η²=0.556; Dunnett’s *post hoc*—Control *vs.* 50 µM: *q*=4.50, *p*=0.0005, *g*=−2.49; Control *vs.* 100 µM: *q*=3.83, *p*=0.0031, *g*=−2.19; other comparisons ns; *n*=5–6/group.) c) Immunofluorescence staining of Ki67 in SH-SY5Y cells after PHCZ exposure. d) Quantification of Ki67-positive cell ratio. (One-way ANOVA: F (5,24) =11.95, *p*<0.0001, η²=0.714; Dunnett’s *post hoc*: Control *vs.* 5 µM: *q*=3.96, *p*=0.0026, *g*=2.04; 10 µM: *q*=4.45, *p*=0.0008, *g*=2.30; 50 µM: *q*=5.66, *p*<0.0001, *g*=2.92; 100 µM: *q*=5.61, *p*<0.0001, *g*=2.89; other comparisons ns; *n*=5/group.) Data was shown as mean ± SEM in b) and d). ns: not significantly different; *: *p*<0.05; **: *p*<0.01.


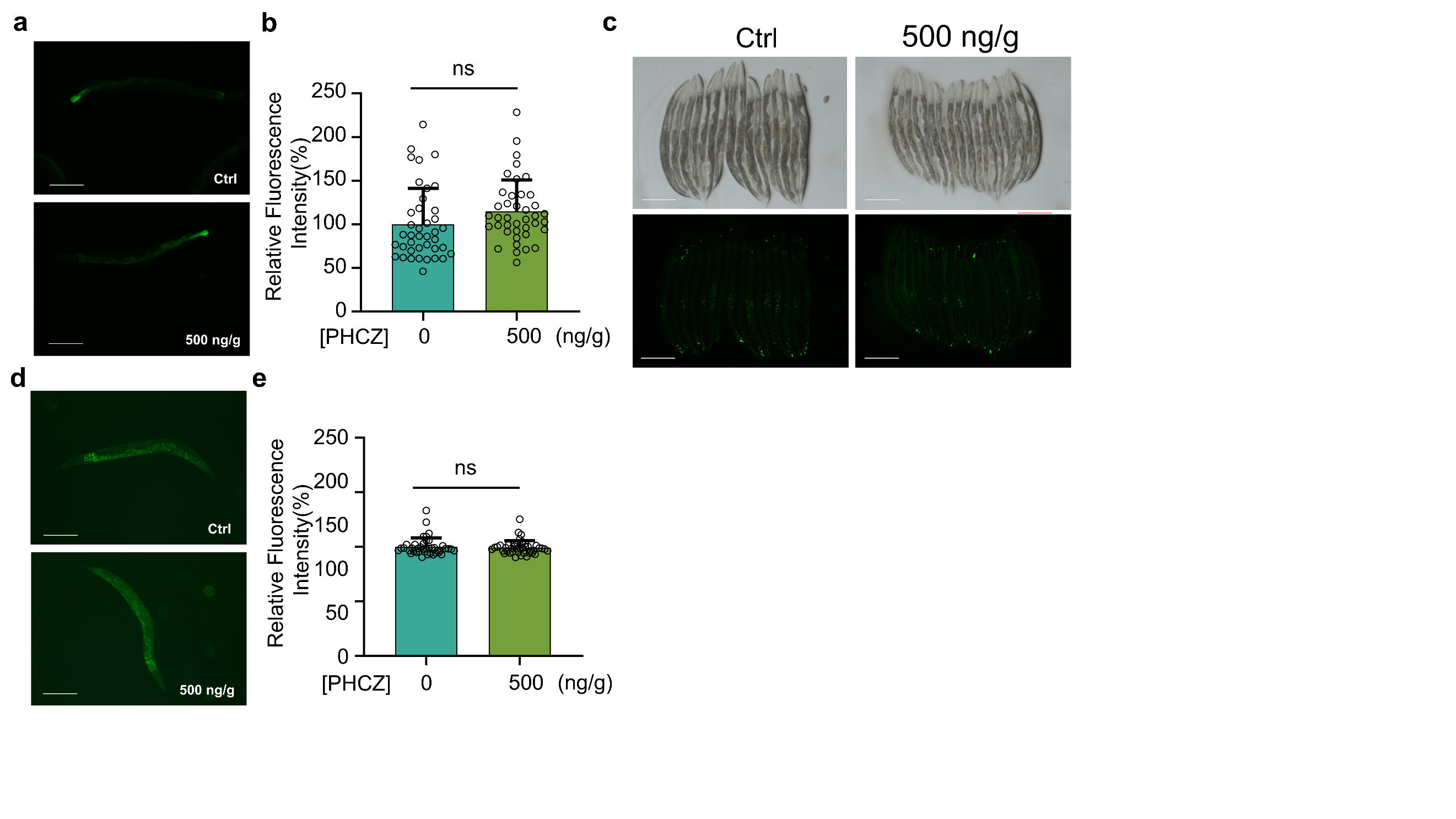


**Figure S4. Unfolded protein response in mitochondria (UPR^mito^) and cytosol (UPR^cyt^); mtDNA amounts *per* cell were not altered upon PHCZ treatment.** a-c) Measurement of UPR^mito^ activation in worms with indicated treatment using fluorescent reporter strains, *P_hsp-6_::GFP* a) and *P_dve-1_::DVE-1::GFP* c), respectively. Quantification of a were shown in b (unpaired *t*, two-sided. *t* (78) =1.716, *p*=0.090 (ns); Δ=14.91 ± 8.69 (95% CI −2.38 to 32.21); η²=0.036; *n*=40/group. Variances not different: *F* (39,39) =1.32, *p*=0.39.)). d-e) Measurement of UPR^cyt^ activation in worms with indicated treatment using fluorescent reporter strains, *P_hsp-16.2_::GFP.* Quantification of d were shown in e (unpaired *t*, two-sided. *t* (80) =0.440, *p*=0.661 (ns); Δ=−0.70 ± 1.59 (95% CI −3.87 to 2.47); η²=0.0024; *n*=41/group. Variances not different: *F* (40,40) =1.605, *p*=0.139). Scale bars represent 50µm in a), c), and d). Data were shown as mean ± SEM in b) and e). ns: not significantly different; *: *p*<0.05; **: *p*<0.01.


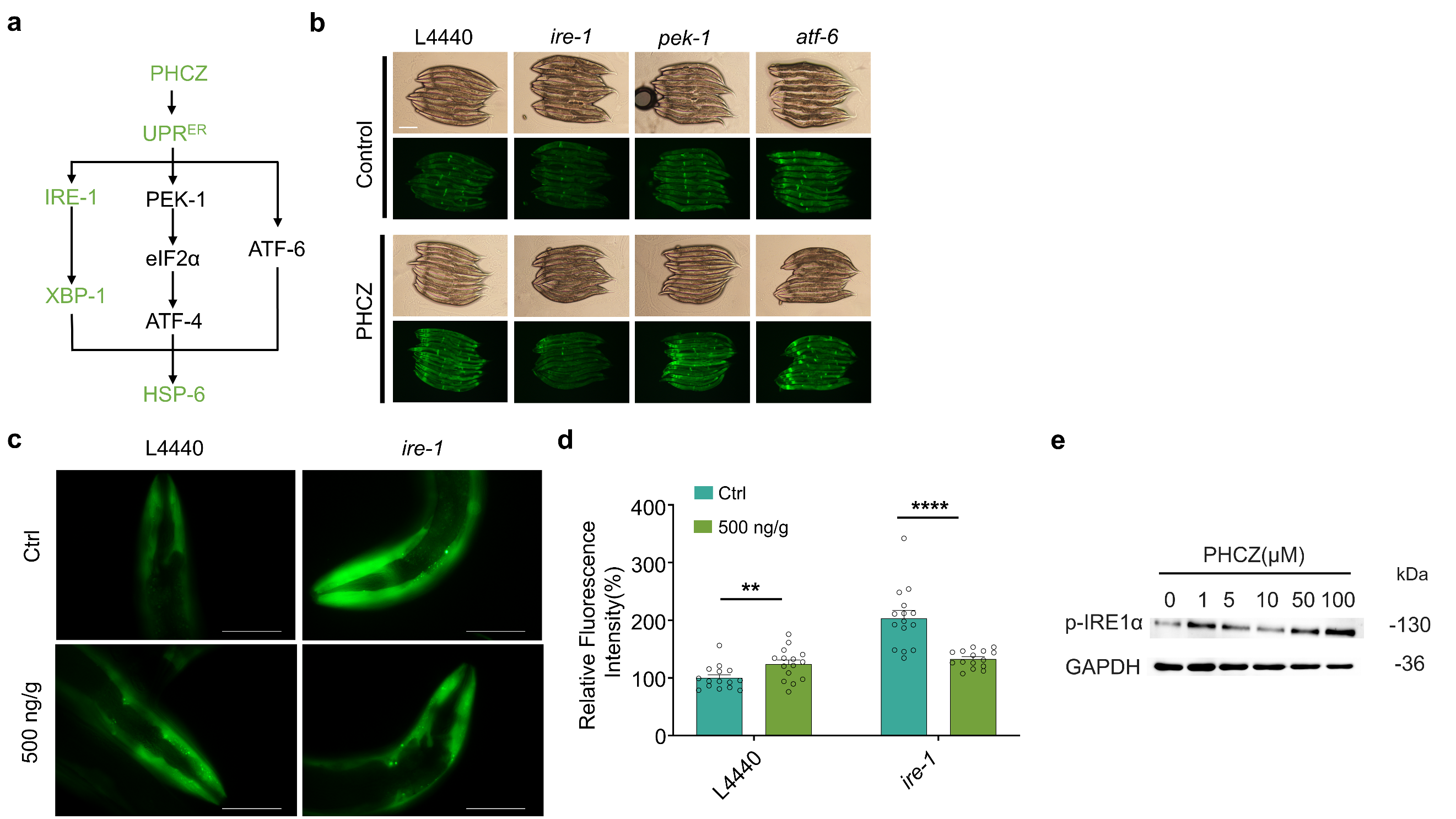


**Figure S5. UPR^ER^ activation in *C.elegans* with indicated RNAi treatments and SH-SY5Y cells.** a) Schematic illustration of the UPR^ER^ regulatory pathways. b) Representative images showing UPR^ER^ activation status, indicated by *hsp-4:gfp* fluorescence. c) Representative images showing distinct states of α-synuclein in *C. elegans* after *ire-1* RNAi. d) Quantification of c) (unpaired *t*, two-sided; BH–FDR correction. L4440: *t* (28) =2.710, *p*=0.0114, *q*=0.00574, Δ=−24.10 ± 8.89; *ire-1*: *t* (28) =4.954, *p*=3.1×10⁻⁵, *q*=3.2×10⁻⁵, Δ=70.45 ± 14.22. *n*=15/group.). e) Western blot analysis of phosphorylated IRE1α in SH-SY5Y cells after PHCZ treatment. Scale bars represent 50 µm in panel b), and 100 µm in panel c). Data were shown as mean ± SEM in d). ns: not significantly different; *: *p*<0.05; **: *p*<0.01; ***: *p*<0.001; ****: *p*<0.0001.


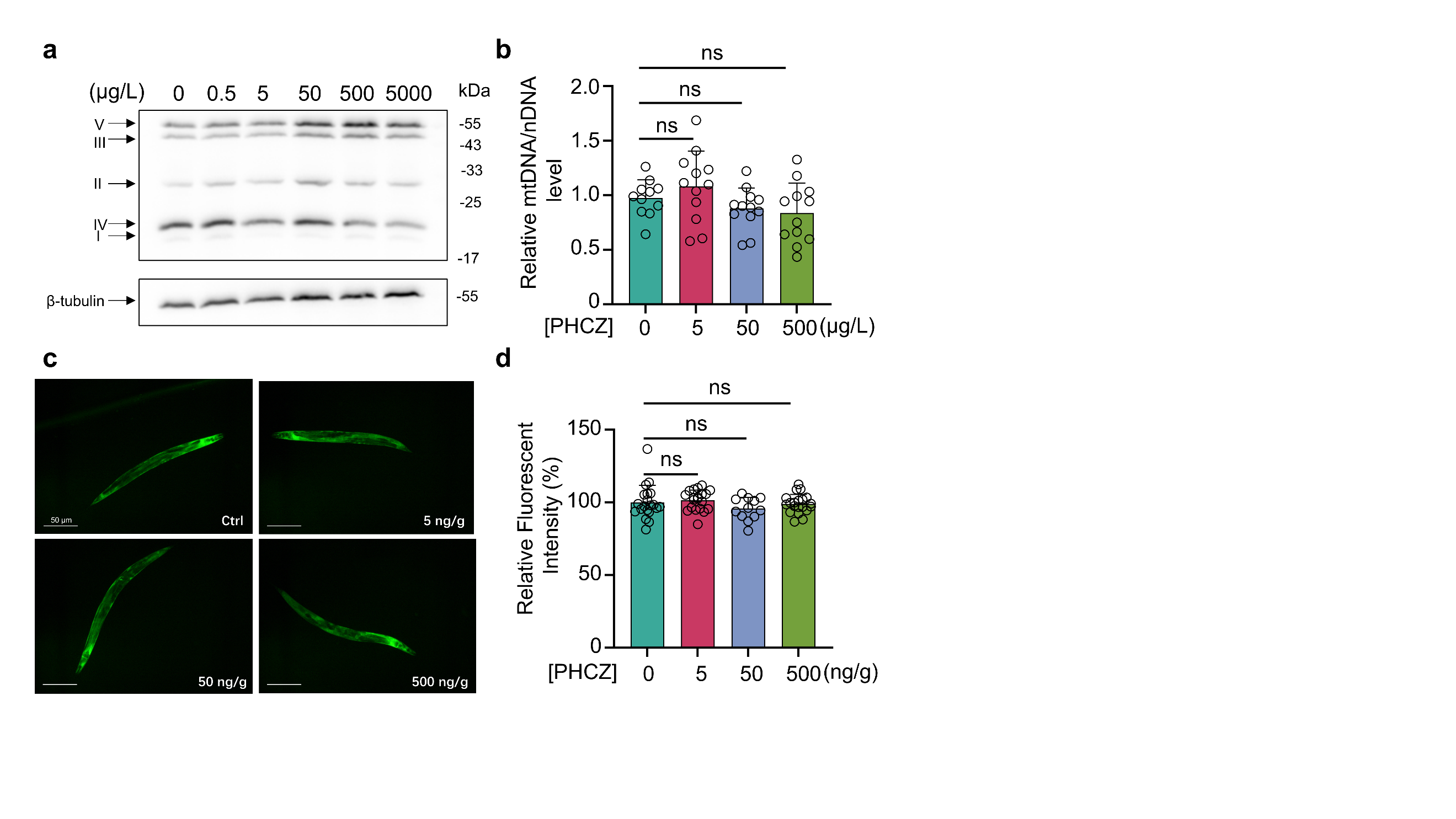


**Figure S6: Components of mitochondrial Electron Transport Chain (ETC) complexes were differentially affected by PHCZ treatment in *C.elegans*, while PHCZ does not activate ROS generation.** a) Quantification of protein levels of ETC complex I, II, III, IV and V in HEK293T cells with treatment of PHCZ at indicated concentrations by western blots. The expected protein sizes are 18kDa (I), 22 kDa (II), 29 kDa (III), 48 kDa (IV), and 54 kDa (V). b) qPCR analysis of mtDNA (tRNA^Leu^ gene encoded in mitochondrial genome) as normalized to nuclei DNA (B2GF-B2GR) in HEK293T cells with indicated treatment. (ANOVA: *F* (3,43) =2.33, *p*=0.0877, η²=0.140. Dunnett’s: Ctrl *vs.* 5 ng/g Δ=−0.107 [−0.359, 0.145], *p*=0.603; Ctrl *vs.* 50 ng/g Δ=0.097 [−0.155, 0.350], *p*=0.665; Ctrl *vs.* 500 ng/g Δ=0.139 [−0.113, 0.391], *p*=0.400. *n*=11–12/group) c) Representative images of ROS level as measured by *gst-4::gfp*. Scale bars represent 50µm. d) Quantification of c (ANOVA: *F* (3,68) =1.21, *p*=0.313, η²=0.0507. Dunnett’s: Ctrl *vs.* 5 ng/g Δ=−1.47 [−8.00, 5.06], *p*=0.911; Ctrl *vs.* 50 ng/g Δ=4.36 [−3.18, 11.91], *p*=0.378; Ctrl *vs.* 500 ng/g Δ=1.02 [−5.52, 7.55], *p*=0.967. *n*=12–20/group.). Data was shown as mean ± SEM in b and d. ns: not significantly different; *: *p*<0.05; **: *p*<0.01.


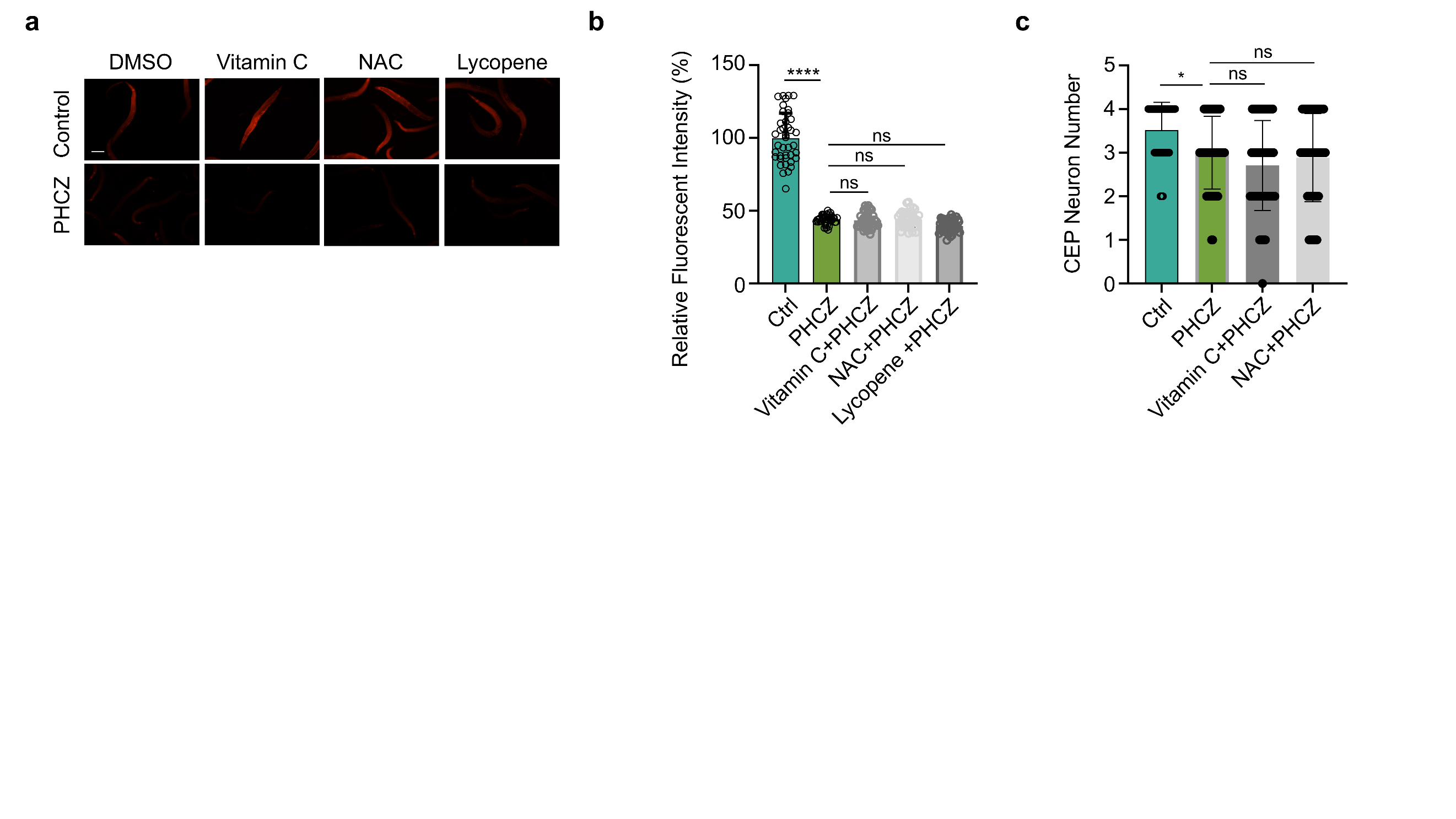


**Figure S7. Antioxidants fail to rescue PHCZ-induced mitochondrial membrane potential loss and dopaminergic neuron loss in *C. elegans*.** a) Antioxidant treatment failed to rescue PHCZ-induced mitochondrial membrane potential loss in *C. elegans*. b) Quantification of relative mitochondrial membrane potential. (One-way ANOVA, F (4,195) =345.5, *p*<0.0001. Tukey’s post hoc: Ctrl *vs.* PHCZ, *p*<0.0001, *g*=6.35; PHCZ *vs.* PHCZ+VC, *p*=0.9980, *g*=0.07; PHCZ *vs.* PHCZ+NAC, *p*>0.9999, *g*=-0.01; PHCZ *vs.* PHCZ+ Lycopene, *p*=0.1569, *g*=0.50. *n*=40/group.) c) Number of CEP neurons in *C. elegans* co-treated with antioxidants and PHCZ. (One-way ANOVA, *F* (3,214) =8.183, *p*<0.0001. Tukey’s post hoc: Ctrl *vs.* PHCZ, *p*=0.0219, *g*=0.56; PHCZ *vs*. PHCZ+VC, *p*=0.3647, *g*=0.34; PHCZ *vs*. PHCZ+NAC, *p*=0.9157, *g*=0.13. *n*=47–62/group.) Data was shown as mean ± SEM in b and c. Scale bars represent 50 µm in panel a. ns: not significantly different; *: *p*<0.05; **: *p*<0.01, ***: *p*<0.001; ****: *p*<0.0001.


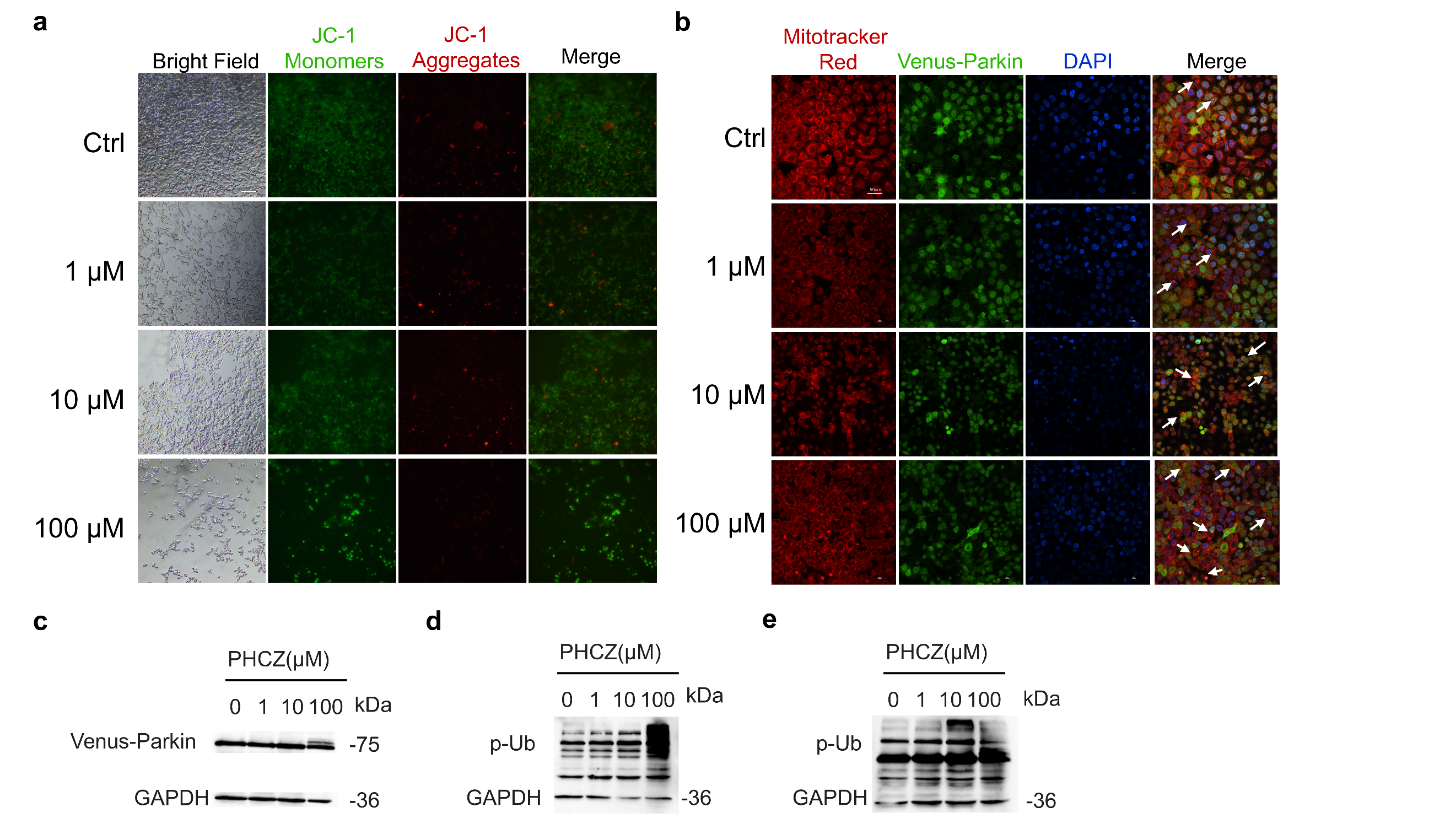


**Figure S8. PHCZ exposure alters mitochondrial membrane potential and activates the PINK1-Parkin pathway in mammalian cells.** a) JC-1 staining of SH-SY5Y cells was used to assess mitochondrial membrane potential after PHCZ treatment. b) Representative images of HeLa-Venus-Parkin cells treated with PHCZ for 24 hours and stained with DAPI and MitoTracker-Red. Hela cells that stably express the fluorescently labeled Parkin (Venus-Parkin) were used in this experiment, because Hela cells have little endogenous Parkin. Parkin recruitment positive cells were indicated with white arrows in the images. c) Western Blot analysis showing phosphorylation of Parkin in HeLa-Venus-Parkin cells after PHCZ treatment. The phosphorylation of parkin causes a molecular weight upshift on the blot. d) Western Blot analysis of phosphorylated polyubiquitinated proteins (smears) in HeLa-Venus-Parkin cells following PHCZ exposure. e) Western Blot analysis of phosphorylated polyubiquitinated proteins (smears) in SH-SY5Y cells treated with PHCZ.


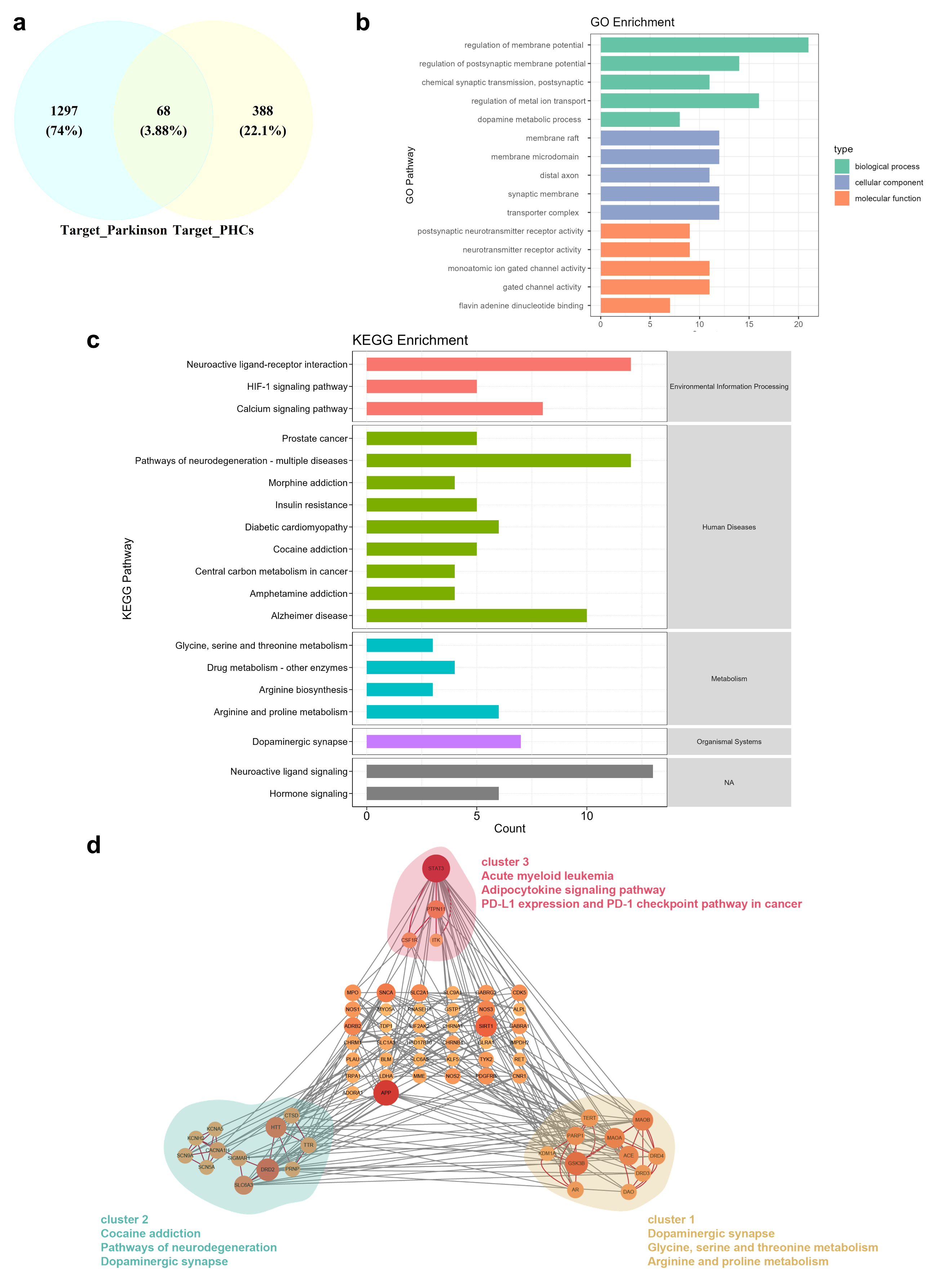


**Figure S9**. **Network toxicology analysis of the association between PHCZ and Parkinson’s disease.** a) Venn diagram illustrating the overlap between PHCZ target genes and Parkinson’s disease-associated genes, highlighting shared protein targets. b) Gene Ontology (GO) enrichment analysis of the intersecting genes. c) Kyoto Encyclopedia of Genes and Genomes (KEGG) pathway enrichment analysis of the intersecting genes. d) Protein-protein interaction (PPI) network of the intersecting genes, visualizing potential mechanistic links between PHCZ exposure and Parkinson’s disease. α-synuclein (SNCA) is shown as the second item from the left in the top row.
